# Targeted N-glycan deletion at the receptor-binding site retains HIV Env NFL trimer integrity and accelerates the elicited antibody response

**DOI:** 10.1371/journal.ppat.1006614

**Published:** 2017-09-13

**Authors:** Viktoriya Dubrovskaya, Javier Guenaga, Natalia de Val, Richard Wilson, Yu Feng, Arlette Movsesyan, Gunilla B. Karlsson Hedestam, Andrew B. Ward, Richard T. Wyatt

**Affiliations:** 1 Department of Immunology and Microbiology, The Scripps Research Institute, La Jolla, California, United States of America; 2 IAVI Neutralizing Center at TSRI, Department of Research and Development, International AIDS Vaccine Initiative, La Jolla, California, United States of America; 3 Department of Integrative Structural and Computational Biology, The Scripps Research Institute, La Jolla, California, United States of America; 4 Department of Microbiology, Tumor and Cell Biology, Karolinska Institutet, Stockholm, Sweden; 5 The Scripps CHAVI-ID, The Scripps Research Institute, La Jolla, California, United States of America; University of Zurich, SWITZERLAND

## Abstract

Extensive shielding by N-glycans on the surface of the HIV envelope glycoproteins (Env) restricts B cell recognition of conserved neutralizing determinants. Elicitation of broadly neutralizing antibodies (bNAbs) in selected HIV-infected individuals reveals that Abs capable of penetrating the glycan shield can be generated by the B cell repertoire. Accordingly, we sought to determine if targeted N-glycan deletion might alter antibody responses to Env. We focused on the conserved CD4 binding site (CD4bs) since this is a known neutralizing determinant that is devoid of glycosylation to allow CD4 receptor engagement, but is ringed by surrounding N-glycans. We selectively deleted potential N-glycan sites (PNGS) proximal to the CD4bs on well-ordered clade C 16055 native flexibly linked (NFL) trimers to potentially increase recognition by naïve B cells *in vivo*. We generated glycan-deleted trimer variants that maintained native-like conformation and stability. Using a panel of CD4bs-directed bNAbs, we demonstrated improved accessibility of the CD4bs on the N-glycan-deleted trimer variants. We showed that pseudoviruses lacking these Env PNGSs were more sensitive to neutralization by CD4bs-specific bNAbs but remained resistant to non-neutralizing mAbs. We performed rabbit immunogenicity experiments using two approaches comparing glycan-deleted to fully glycosylated NFL trimers. The first was to delete 4 PNGS sites and then boost with fully glycosylated Env; the second was to delete 4 sites and gradually re-introduce these N-glycans in subsequent boosts. We demonstrated that the 16055 PNGS-deleted trimers more rapidly elicited serum antibodies that more potently neutralized the CD4bs-proximal-PNGS-deleted viruses in a statistically significant manner and strongly trended towards increased neutralization of fully glycosylated autologous virus. This approach elicited serum IgG capable of cross-neutralizing selected tier 2 viruses lacking N-glycans at residue N276 (natural or engineered), indicating that PNGS deletion of well-ordered trimers is a promising strategy to prime B cell responses to this conserved neutralizing determinant.

## Author summary

A major challenge in HIV-1 vaccine design is to generate antibodies directed toward conserved broadly neutralizing epitopes on the surface-exposed viral envelope glycoprotein (Env). Most conserved epitopes are masked by self N-glycans, limiting naïve B cell recognition of the underlying protein surface following Env vaccination or during natural infection. Recently, soluble faithful mimics of the HIV Env spike have been developed, but their capacity to elicit broadly cross-reactive tier 2 (clinical isolate) neutralizing responses is limited. The conserved primary receptor, CD4 binding site, is a known neutralizing determinant, but is flanked by self-N-linked glycans, limiting Ab access to this site. Here, we removed up to four N-glycans surrounding the CD4 binding site without affecting trimer stability and conformation as demonstrated by multiple biophysical methods. Using these well-ordered trimers, we performed an immunogenicity experiment, demonstrating that glycan-deleted trimers elicited superior neutralizing responses compared to the fully glycosylated trimers, resulting in detectable cross-neutralization of a subset of tier 2-like viruses.

## Introduction

The HIV-1 envelope glycoprotein (Env) trimer is the sole target for neutralizing antibodies on the surface of the virus, mediating both receptor attachment and entry. Recently, high resolution structures of the native and native-like HIV-1 trimer revealed the extensive N-linked glycan shielding that has evolved to protect most of the underlying polypeptide surface from access by B cells and most antibodies [1–4]. However, the past decade has identified multiple broadly neutralizing antibodies (bNAbs) from selected HIV-infected individuals [5], demonstrating that the human immune system can elicit antibody responses that can penetrate and, in some cases, recognize the glycan shield. These studies reveal several cross-neutralizing epitopes, including those localized to the gp120 V2 apex [6–10], the V3-proximal N332 super site [10,11], the CD4 binding site [12–16], the gp120-gp41 interface site [17–20] and membrane proximal external region (MPER)-directed site [11,20].

Antibody selection pressure to HIV-1 has evolved considerable host-derived N-glycan masking, occluding most conserved potential neutralizing determinants. Multiple bNAbs isolated from chronic HIV-1 patients are directed against the HIV-1 Env conserved primary CD4 receptor-binding site (CD4bs) [12–16]. The CD4bs surface itself is devoid of N-linked glycosylation but is shrouded by N-glycans around its periphery. Presumably, the shielding restricts antibody access but is sufficient to allow the critical function of CD4 receptor engagement to initiate viral entry [21–27]. Therefore, in this study we sought to determine if trimers with targeted N-glycan deletion would more efficiently activate B cells and better elicit neutralizing antibodies. Since the CD4bs is partially accessible, we selected this site to test targeted N-glycan deletion to prime B cell responses and neutralizing antibodies.

**Fig 1:**
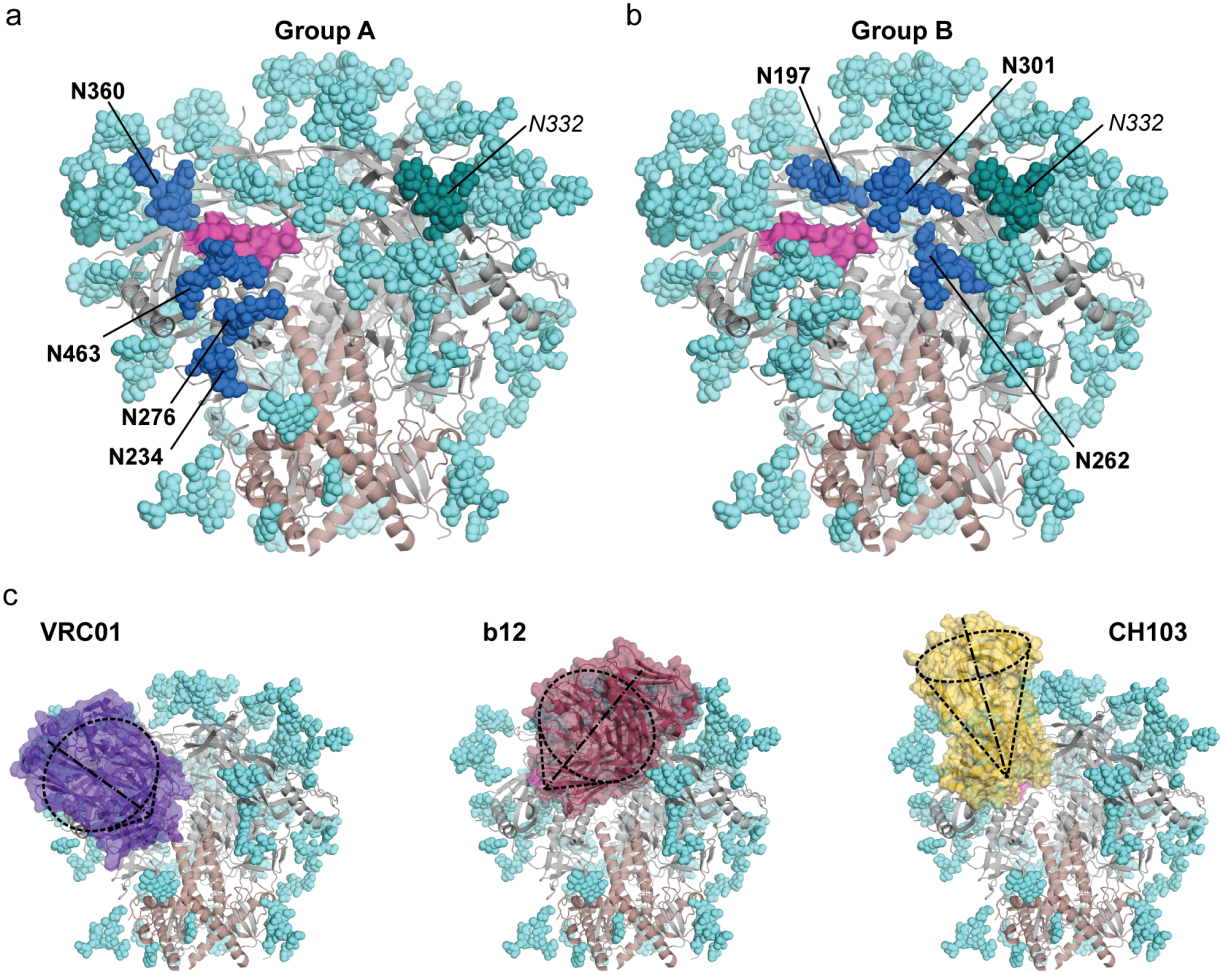
HIV Env trimer N-glycans and the CD4bs. Structure of soluble BG505 SOSIP.664 HIV trimer (PDB accession number 5FYL) with gp120 in gray, gp41 in brown and the CD4bs shown as a magenta surface. N-glycans are shown in shades of blue. (a) The Group A N-glycans proximal to the CD4bs are shown in dark blue as indicated in bold and the N332 N-glycan is shown in dark turquoise. (b) The Group B N-glycans proximal to the CD4bs are shown in dark blue and are indicated in bold. N332 N-glycan is shown in dark turquoise. (c) Trimer docking models of VRC01 (purple), b12 (red) and CH103 (yellow) Fabs, each approaching the CD4bs with different angles of access.

The known CD4bs-directed bNAbs isolated from chronically infected individuals are divided into two major classes depending upon their mode of recognition of the CD4bs and their VH family usage [14]. One class is comprised of the variable heavy (VH)-restricted bNAbs that include the VRC01-class antibodies. These VRC01-like antibodies use the VH1-2*02 or VH1-46 heavy chain gene segments and contact the CD4bs primarily with complementarity determining region 2 (HCDR2)-encoded residues and are less dependent on the HCDR3 than most antibodies [15,28]. The light chains of these bNAbs, usually kappa, also display common properties by possessing relatively short or flexible CDRs, often a 5 amino acid LCDR3. The second class of CD4bs-directed bNAbs are not VH-restricted and use their diverse HCDR3s to contact the CD4bs [22,23]. The bNAbs from both classes bind the CD4bs with roughly similar lateral angles of approach (Fig 1), which is associated with their breadth and potency [28]. Other CD4bs-directed monoclonal antibodies (mAbs), which are broad but less so than the VRC01-like class, such as CH103 [23] or b12 [29], display more vertical or less optimal angles of approach to the CD4bs. Presumably, a major restriction for activation of both of these classes of bNAbs is efficient engagement of the corresponding naïve B cell receptors. Access to the CD4bs is limited due to its recessed location, obstructed by extensive glycan shielding and tight quaternary packing of the antibody-selected Env trimeric spike [30]. The conservation and the fact that N-linked glycans are not part of the contact surface [31], along with the isolation of multiple bNAbs against this site from several HIV-infected patients, elevates the CD4bs as an attractive target for HIV-1 vaccine design.

Here, guided by CD4bs antibodies and Env structures [2,32,33], we selectively deleted potential N-linked glycans (PNGS) proximal to the CD4bs on the well-ordered soluble clade C 16055 NFL TD CC trimers because of their high degree of stability and homogeneity [33,34]. Our goal was to enhance *in vivo* engagement by naïve B cells specific for this conserved neutralizing determinant regardless of the genetic properties of their B cell receptors (BCRs), similar in concept to two recent studies performed in parallel [35,36]. We generated a series of N-glycan-deleted variant trimers that maintained native-like trimer conformation without significant loss in stability. We used a panel of CD4bs-directed bNAbs from both the VH gene-restricted and the CDRH3-using classes that demonstrated better accessibility of the CD4bs on N-glycan-deleted trimer variants, while maintaining conformational or steric occlusion defined by the trimer quaternary structure. We also introduced a subset of the PNGS-deletions into the full-length 16055 Env to generate pseudoviruses, and demonstrated that they retained resistance to non-neutralizing mAbs. We performed rabbit immunogenicity experiments using two approaches comparing glycan-deleted to fully glycosylated NFL trimers. The first was to delete four PNGS sites and then boost with fully glycosylated Env; the second was to delete the four sites and gradually re-introduce these N-glycans in subsequent boosts, an approach previously not yet tested in the context of native-like trimers. These experiments revealed that the PNGS-deleted trimers more rapidly elicited neutralizing antibodies for CD4bs-PNGS-deleted viruses and more potent responses against fully glycosylated wt virus. We demonstrated that part of this activity was CD4bs-directed and could be boosted with fully glycosylated trimers to elicit weak but detectable cross-neutralization. The analysis presented here indicates that targeted N-glycan deletions is a promising approach to more efficiently elicit antibodies directed toward the conserved CD4bs.

## Results

### NFL trimers with selected N-glycan deletions retain a native-like conformation

To preferentially increase recognition of the gp120 CD4bs, while maintaining well-ordered trimeric native-like structure, we selected a highly stable and homogeneous soluble trimer 16055 NFL TD CC (T569G), as the parental backbone for targeted N-glycan deletions, designated as “PT” for “Parental Trimer” for the remainder of this manuscript. This soluble trimeric protein is derived from an Indian subtype C HIV-1 Env sequence that was isolated from a patient following acute infection [37]. The original NFL trimer design [38] consists of a 10 residue (G_4_S) flexible linker between the REKR-deleted Env gp120 C-terminus and the unmodified gp41 N-terminus, contains a I559P mutation in gp41 and is truncated at residue 664. The NFL TD, for trimer-derived, possesses substitutions at residues E47D, K49E, V65K, E106T, I165L, E429R, R432Q, A500R to increase trimer formation and stability [34] and a T569G substitution that increases homogeneity and yields [33]. An engineered intra-protomer disulfide I201C-A433C (CC) prevents CD4-induced conformational rearrangements that expose non-neutralizing determinants [34,39].

Guided by Env trimer structures [2,32,40], we deduced that several N-linked glycosylation sites occlude the gp120 CD4bs within the quaternary packing of trimer (Fig 1a and 1b). In addition, by inspecting the angles of access determined for several CD4bs-directed bNAbs [14,23,41,42], we reasoned that deleting one set of PNGSs, by genetic alteration of this motif, would increase access for most bNAbs approaching the CD4bs with a VRC01-like lateral path (Group A, Fig 1a) without allowing access by non-broadly neutralizing CD4bs-directed mAbs such as F105. Although we included the VRC01-like antibodies as design guides, we also included the non-VH-gene-restricted class of CD4bs-directed bNAbs such as VRC13 or VRC18 [43], with the objective to open access to the CD4bs unfettered by VH or VL gene-restricted requirements. The PNGSs revealed by this analysis include N234, N276, N360 and N463 amongst others (Group A, Fig 1a). We chose to not alter PNGSs at the V-cap trimer apex (i.e. N386) because we showed previously that non-broadly neutralizing CD4bs-directed mAbs bind this region by a vertical angle that allows access to the CD4bs on some tier 1 viruses (HXBc2), that is occluded by N-glycans on tier 2 viruses [44]. We also determined that deletion of the additional N-glycans N197, N262 and N301 would potentially open access to the CD4bs for antibodies displaying a similar angle of approach as the bNAb, b12 (Group B; Fig 1b).

Following lectin purification, we analyzed trimer production by size-exclusion chromatography (SEC) relative to the PT as the first criterion to assess PNGS-deleted trimer integrity. Single (A1, B1), double (A2) and triple (A3) glycan-deleted trimer variants were analyzed (S1 Fig). In parallel, we investigated the conformational state of the selected glycan-deleted variants by negative stain EM as a second criterion to assess PNGS-deleted trimer integrity (S1 Fig). As a third criterion, we analyzed trimer stability and homogeneity by DSC to assess trimer integrity harboring the targeted genetic PNGS deletions (S2 Fig). These biophysical analyses are detailed in the Supplementary materials and our findings can be summarized as follows. We determined that mutations N276Q, N301Q and the combinations of mutations N276Q/N360Q, N276Q/N463Q and N276Q/N360Q/N463Q minimally affected the trimer yields and thermostability and allowed native-like trimer conformation (S1 and S2 Figs). On the other hand, the PNGS mutations N197Q, N234Q and N262Q affected trimer integrity. Deletion of N262 PNGS resulted in extremely low trimer expression (S1 Fig). Similar effects were observed when N234Q was introduced in the combination with mutations N276Q and N463Q (S1 Fig). In the case of the N197Q substitution, we observed a substantial loss of both the propensity to form well-ordered trimers and protein thermostability (S2 Fig). Therefore, we further focused our analysis on PNGS modifications that did not affect trimer integrity, namely, N276Q, N301, N360Q, and N463Q.

**Fig 2:**
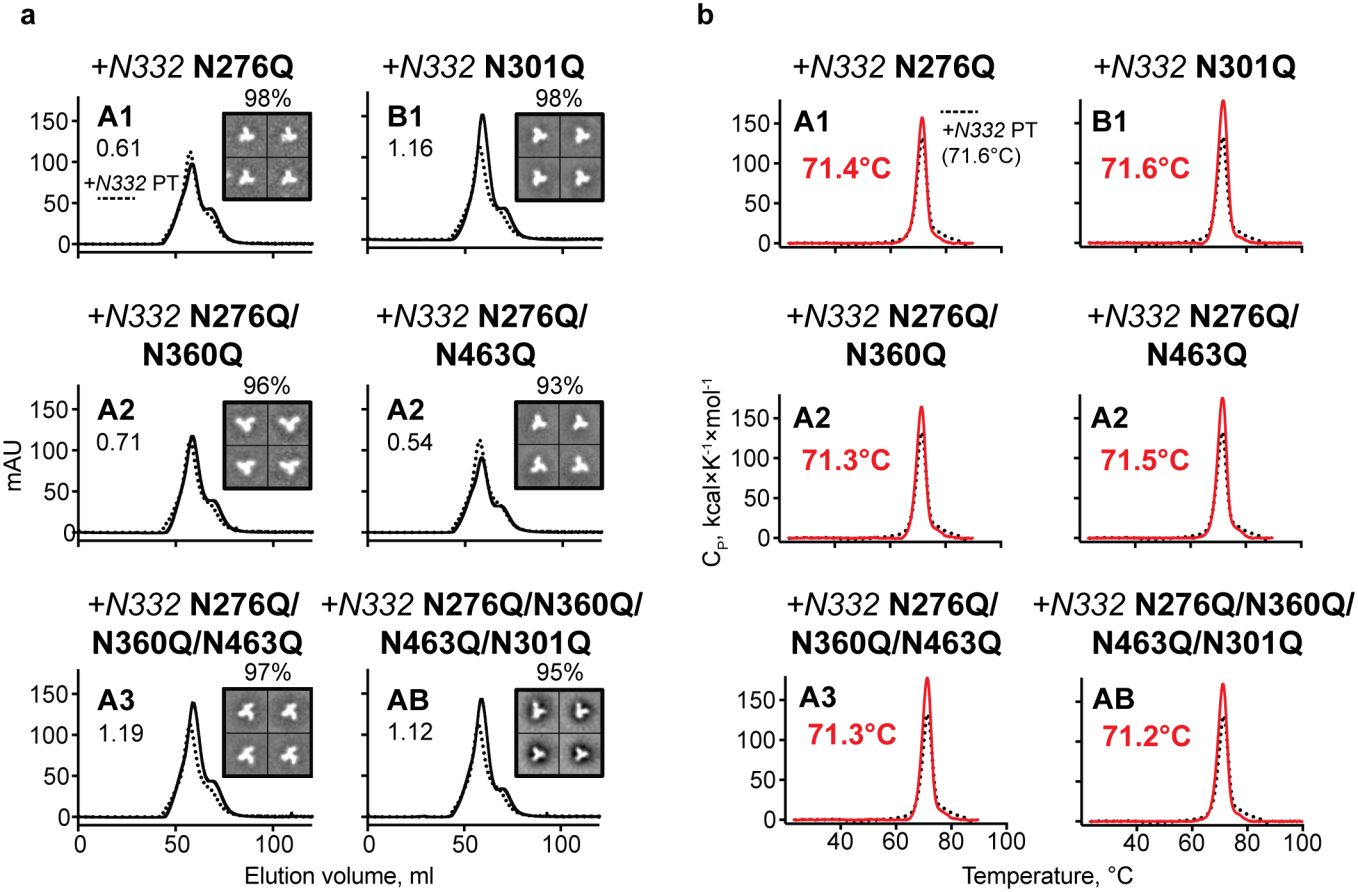
Characterization of lectin affinity-purified 16055 glycan-deleted trimers with the 332 N-glycan restored. (a) SEC profiles and EM 2D class averages. A1 or B1, A2, A3 and AB indicate trimers with one, two, three and four N-glycan deletions, respectively. SEC profiles of N-glycan-deleted trimers (solid line) are shown in comparison with the *+N332* PT trimer (dotted line) and the expression level relative to expression level of *+N332* PT is shown on each SEC graph. Percentage of native-like trimers is indicated above the 2D class averages representative images. (b) DSC thermal transition curves and derived T_m_s of glycan-deleted trimers (red solid line) compared to the backbone glycoprotein *+N332* PT (black dotted line).

As mentioned above, the 16055 Env naturally lacks a PNGS at residue N332, located in the gp120 outer domain. However, this N-glycan site is generally well-conserved across HIV Env strains and is central to the 332N-glycan “supersite” that is the target of many bNAbs such as 2G12, PGT128 and PGT135 [27,45]. We reasoned that, in addition to restoring an important neutralizing determinant, that genetic restoration of this N-glycan might impact overall trimer stability, thereby allowing us to delete additional PNGS from Group A (Fig 1a). Accordingly, we introduced the PNGS at residue 332 in the 16055 PT by a K334S mutation. We termed this N332-glycan-restored trimer as “+*N332* PT”, where the italicized *N* refers to the N-glycan, not the asparagine residue common to both trimer-types. To confirm conformational integrity, we compared the thermal transition midpoints (T_m_s) and the EM 2D class averages for the two trimeric proteins with and without the PNGS at residue 332 (S3a and S3b Fig). The *+N332* PT trimer was minimally more stable than the isogenic PT lacking the 332 N-glycan, displaying a T_m_ increase of +0.3°C (S3a Fig). EM analysis showed nearly identical populations of native-like trimers for both proteins. We demonstrated that there were no significant differences for the binding by a panel of CD4bs-directed mAbs (S3c Fig) and no difference in binding by the trimer-preferring bNAbs, PGT145, PG9 and PG16. Restoration of the N332 supersite was confirmed by efficient binding by the bNAbs, PGT135 and PGT128 (S3c Fig). Expression and yields of the PNGS-deleted NFL trimeric proteins for both Group A and B were not affected by the glycan alterations and EM analysis revealed that the trimeric glycoproteins retained a native-like conformation (Fig 2). DSC analysis of both sets of N-glycan deleted trimers showed that their T_m_s remained practically identical suggesting that the N-glycan alterations did not affect stability of the proteins (Fig 2B). These analyses allowed us to select the best combination of N-glycan deletions proximal to the CD4bs in the native-like NFL context.

### Deletion of N-glycans proximal to the CD4bs enhances Env recognition by selected CD4bs-directed bNAbs

To examine the effects of N-glycan deletion on antibody accessibility at the CD4bs, we analyzed binding of a set of CD4bs-directed bNAbs to specific N-glycan-deleted variants compared to their respective parental trimers. For this analysis, we used a His-capture ELISA, to maintain native-like trimer confirmation to assess bNAb recognition as previously described [34]. Preservation of a native-like trimer conformation was confirmed by efficient recognition by the trimer-dependent bNAb, PGT145 (S5 and S6 Figs) [46], and by poor recognition by the non-broadly neutralizing, CD4bs-directed mAb, F105 [47].

**Fig 3:**
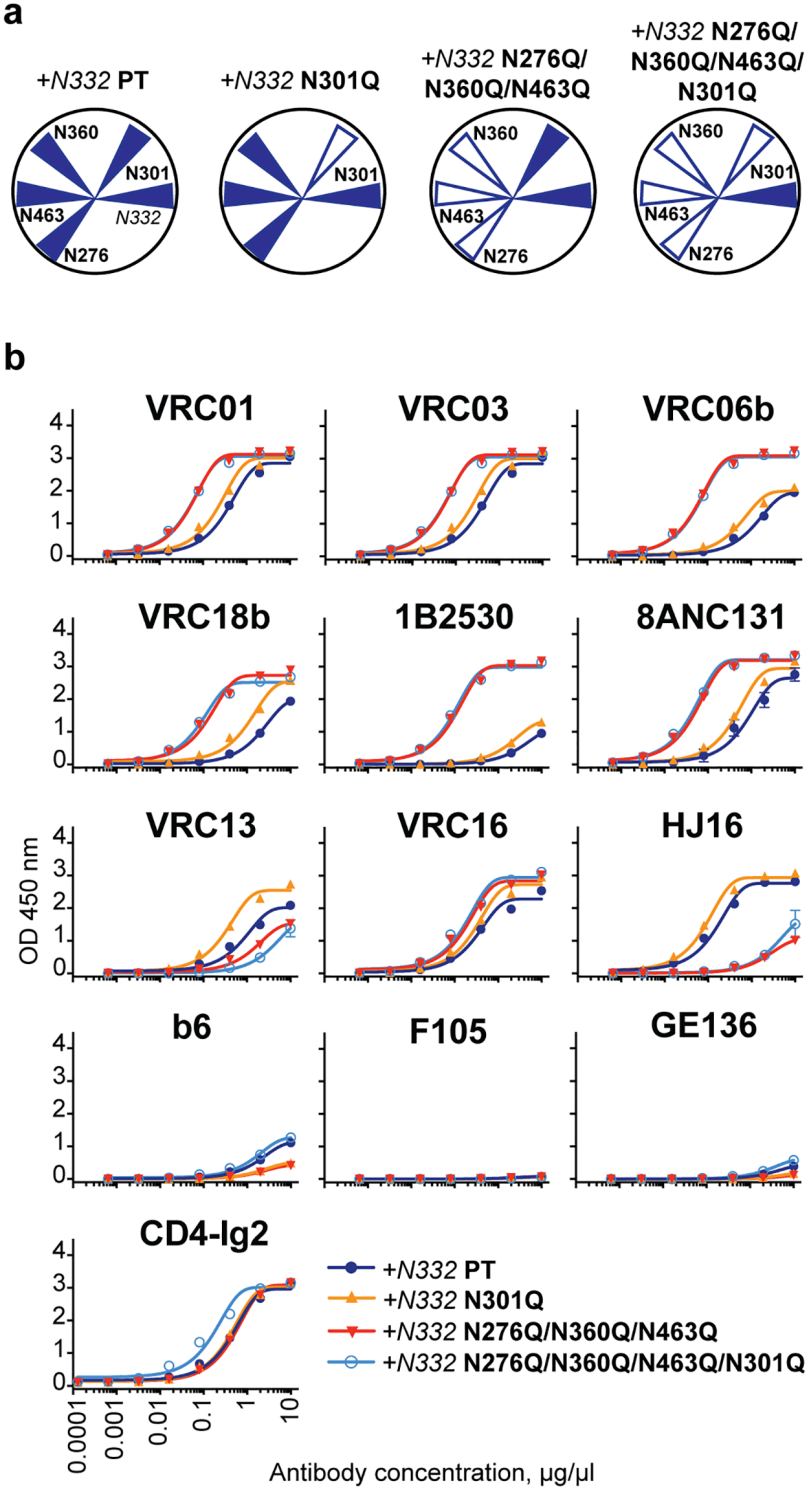
CD4bs-specific antibody binding profiles to the N-glycan deleted trimers. (a) Schematic presentation of N-glycan composition around the trimer CD4bs in the selected N-glycan-deleted trimers. Filled blue triangle—the N-glycan is present; empty blue triangles—the N-glycan is genetically deleted. (b) Comparison of the *+N332* PT (dark blue) with *+N332* N301Q (yellow), *+N332* N276Q/N360Q/N463 (red) and *+N332* N276Q/N360Q/N463/N301Q (light blue) trimers. Recognition of His-captured trimers by the trimer-elicited rabbit serum were analyzed in duplicate at each antibody dilution. The error bars indicate variance of the mean binding values (OD450 nm) and a representative experiment of three independent repeats is shown.

We selected a panel of monoclonal antibodies based on their differential ability to neutralize 16055 pseudovirus and their different modes of Env recognition. Access to the CD4bs was assessed to determine whether specific targeted N-glycan deletions rendered this region more accessible for mAbs of different origin, angles of approach and neutralizing capacity. We demonstrated increased binding by the bNAbs VRC01, VRC03, VRC06b, VRC18b (VH1-2-derived; [12,14,48]) and 1B2530 and 8ANC131 (VH1-46-derived; [24]) to the N276Q/N463Q glycan-deleted variants with or without N332 restored (Fig 3, S4 and S6 Figs). Increased binding by the bNAbs VRC01, VRC03, VRC06b, VRC18b, 1B2530 and 8ANC131 was also detected to the *+N332* N276Q/N360Q/N463Q and *+N332* N276Q/N360Q/N463Q/N301Q triple and quadruple N-glycan-deleted variants compared to the fully glycosylated +*N332* PT backbone (Fig 3, S6 Fig). For the *+N332* N301Q glycan-deleted variant, the difference in binding was less pronounced (Fig 3, S6 Fig).

We next assessed recognition by the set of HCDR3-using CD4bs-directed mAbs, VRC13, VRC16 and HJ16. Binding to the *+N332* N301Q glycan-deleted variant was enhanced in comparison with +*N332* PT for all three antibodies (Fig 3, S6 Fig). As expected, HJ16 binding was impaired when the PNGS at residue 276 was altered, consistent with its known (Fig 3, S4 and S6 Figs) N276 glycan-dependence [49]. VRC13 recognition was similarly impaired by deletion of the N463 PNGS and is likely dependent upon the presence of this N-glycan for efficient Env recognition (Fig 3, S6 Fig). Both of these changes in recognition are consistent with deletion of the N-glycans at residues 276 and 463 by altering PNGS motif. With the four N-glycans eliminated in the 16055 trimers, we tested binding by the germline-reverted antibodies VRC01gl, VRC13gl, VRC16gl but as expected, did not detect binding (S5 and S6 Figs).

To complete the antigenic analysis of the N-glycan-deleted trimer variants, we detected efficient recognition by the trimer-preferring V2-apex-directed bNAbs, PG9 and PG16, confirming that the trimer native-like conformation was not affected by the N-glycan deletions (S5 and S6 Figs). No binding differences were observed for the N332-glycan “supersite” antibodies PGT121 and PGT135, whereas, 2G12 [11] displayed slightly decreased recognition for the 301 N-glycan-deleted trimer variants (S5b and S6 Figs).

In sum, targeted N-glycan deletions preferentially enhanced antibody recognition by the majority of CD4bs-directed antibodies without significantly altering bNAb recognition of other Env regions.

### Bio-layer interferometry (BLI) confirms enhanced binding of the N-glycan-deleted trimer by the CD4bs-directed bNAb, VRC03

**Fig 4:**
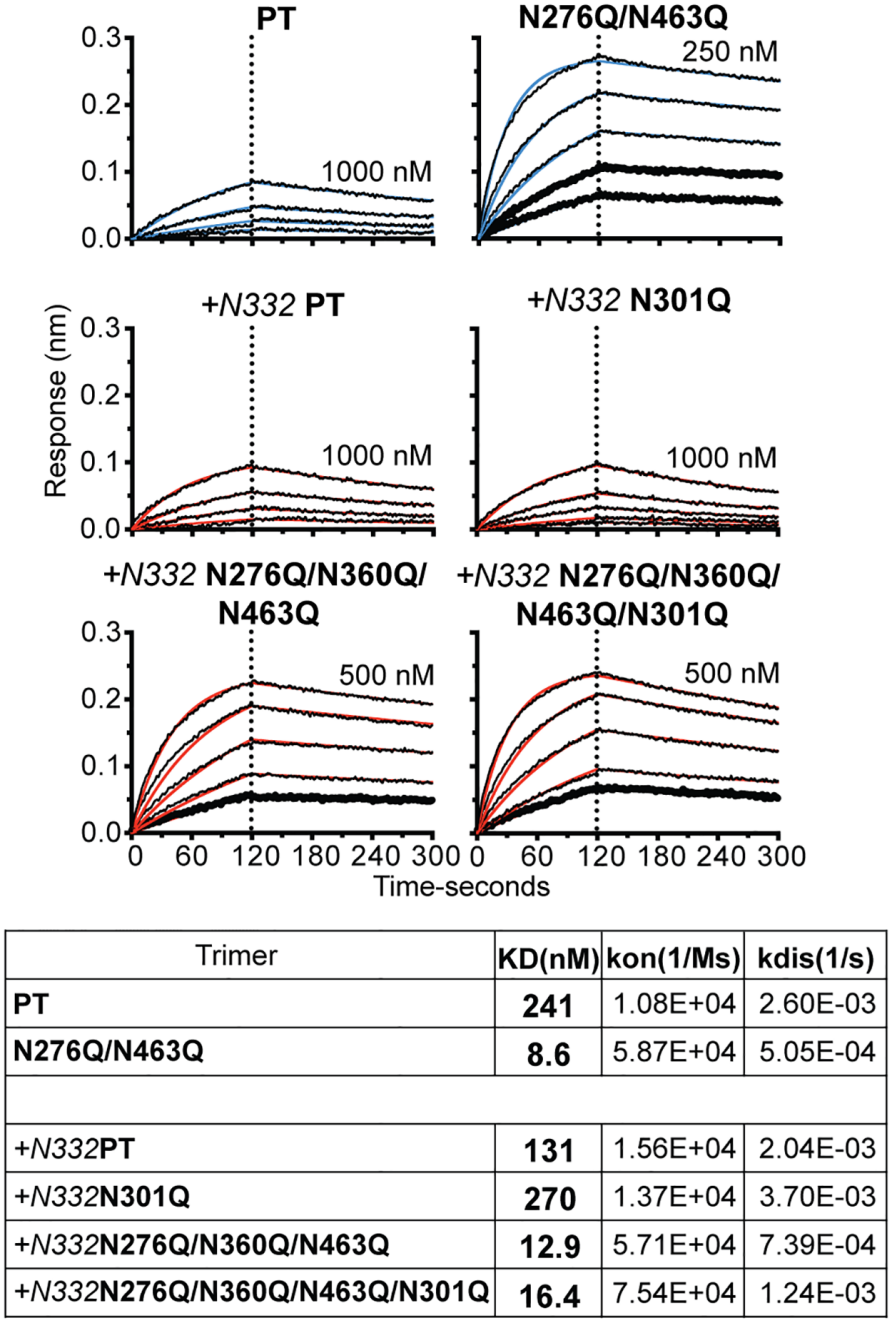
Binding kinetics for glycan deleted trimers with the VRC03 Fab. Bio-layer interferometry (BLI) curves were generated with the PT and N276Q/N463 trimers (blue fitted curves) and *+N332* PT with *+N332* N301Q, *+N332* N276Q/N360Q/N463 and *+N332* N276Q/N360Q/N463/N301Q trimers (red fitted curves) immobilized on an anti-His sensor with serial dilutions of the VRC03 Fab at the concentrations indicated. A tabular summary of the K_d_, k_on_ and k_off_ is shown.

Next, we used BLI (Octet) to assess the effect of N-glycan deletion on the binding efficiency of the CD4bs-directed bNAb, VRC03. Since the bivalent VRC03 IgG can potentially bind CD4bs epitopes on multiple trimers, creating avidity, we generated the VRC03 Fab to permit precise determination of the affinity of this interaction with trimer. Using the Fab as the monomeric analyte in solution, we found that the N276Q/N463Q trimer, when captured in the sensor surface, was recognized by the VRC03 Fab approximately 30-times more efficiently compared to the PT “backbone” trimer (Fig 4). In case of glycan-deleted variants of +*N332* PT, there was a 10- and 8-fold difference, respectively, in affinity for the *+N332* N276Q/N360Q/N463Q and *+N332* N276Q/N360Q/N463Q/N301Q variants compared to the backbone protein. The binding of *+N332* N301Q variant was two-fold lower in comparison with the +*N332* PT backbone.

Following the detected increase in VRC03 Fab affinity for the four-position N-glycan-deleted trimer, we assessed the effect of this N-glycan deletion on stoichiometry by negative-stain EM. We generated complexes and obtained 2D class averages and 3D reconstructions of the *+N332* N276Q/N360Q/N463Q/N301Q variant compared to the backbone +*N332* PT trimer. We found that despite the large affinity increase of VRC03 Fab for the N-glycan-deleted trimer detected by BLI (and ELISA), the stoichiometry of the interaction was not altered relative to the +*N332* PT backbone as determined by EM (S7 Fig).

### Full-length 16055 Env pseudoviruses with CD4bs-proximal PNGS deletions retain a “tier 2-like” phenotype

**Fig 5:**
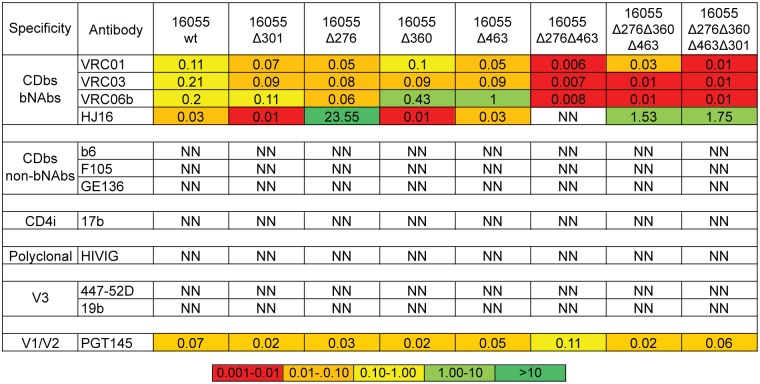
Antibody sensitivity of glycan-deleted variants of 16055 pseudovirus. Neutralization IC_50_ values of the panel of bNAbs and mAbs are shown and color-coded for concentrations (μg/ml) regarding potency as indicated. NN = No Neutralization. These experiments were performed two independent times for the antibodies shown.

To evaluate Ab responses elicited by the PNGS-deleted trimer immunogens, we generated full-length 16055 Env expression plasmids encoding matching CD4bs-proximal N-glycan deletions. We generated 16055 HIV-1 pseudoviruses that we named “wt” for the fully glycosylated Env and “Δ followed by a numeral” to specify N-glycan deletions at the stated Env positions and assessed their properties of entry and neutralization sensitivity. For example, a pseudovirus with Env possessing two N-glycan deletions at positions 276 and 463 is designated 16055Δ276Δ463. Consistent with the observations made for the soluble Env trimers, pseudoviruses lacking two to four N-glycans were more sensitive to neutralization by VRC01, VRC03 and VRC06b and, as expected, less sensitive to the N276-glycan-dependent bNAb, HJ16 (Fig 5). In the 16055 virus context, each of the glycan-deleted pseudoviruses displayed a tier 2-like phenotype as defined by selected mAbs and HIVIG (HIV Immunoglobulin, lot# 140406). In particular, deletion of the N-glycan residue N301 often causes a”global opening” or tier 1 phenotype for other pseudoviruses with this same mutation (i.e., YU2, JRFL and SS1196) [50,51], but it did not cause the same effect in the 16055 context. All 16055 pseudoviruses deleted of their Env CD4bs-proximal PNGS remained insensitive to the non-neutralizing mAbs, b6, F105, GE136, 17b, 447-52D and 19b (Fig 5), as well as to polyclonal HIVIG derived from a pool of HIV-infected individuals. This analysis indicated that the same N-glycan deletions that were tolerated in the context of soluble PT and +*N332* PT proteins also did not affect the native Env conformation on the pseudovirus, while increasing bNAb access to the CD4bs (Fig 5). We observed that the pseudovirus 16055Δ276Δ463 was the most sensitive to the CD4bs-directed bNAbs, and less sensitive to PGT145, in comparison with other N-glycan-deleted viruses, even those variants with additional N-glycan modifications.

This set of Env N-glycan-modified pseudoviruses recapitulated the trimer antigenic profiling of our N-glycan-deleted soluble trimers and represents a useful set of tools to characterize antibody responses generated by such trimers.

### Immunization with N-glycan-deleted trimers generates more rapid and consistent HIV-1 neutralizing antibody responses compared to unmodified trimers

**Fig 6:**
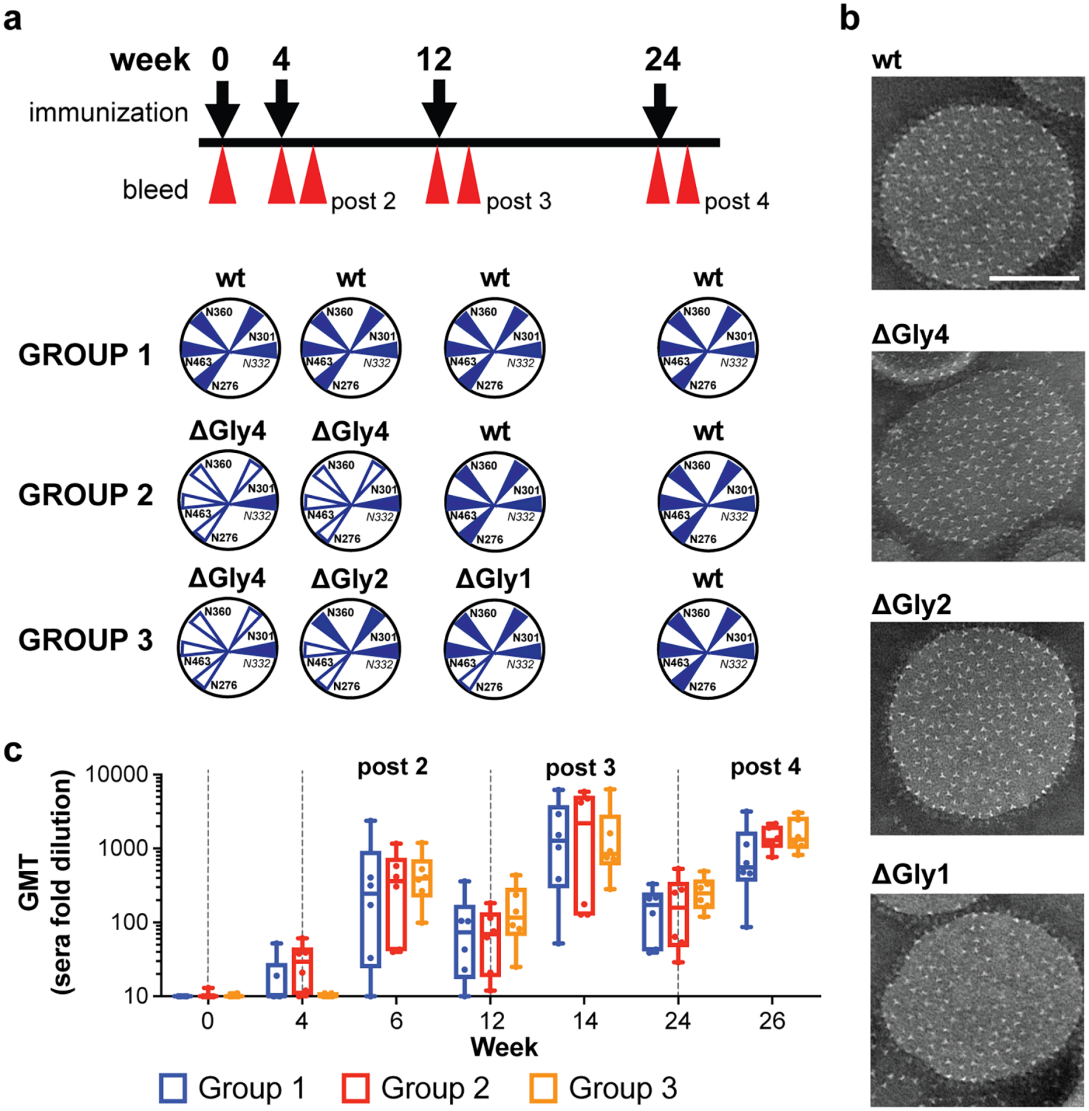
Immunogenicity of glycan-deleted trimers. (a) The immunogenicity regimen and respective immunogens for Groups 1, 2 and 3 are shown. In brief, rabbits were immunized at weeks 0, 4, 12 and 24. Test bleeds are indicated by the red arrows following each immunization. (b) Representative negative stain EM images of the liposomes coupled with the respective trimers. The white scale bar on the top wt trimer-liposomes image is equivalent to 100 nm. (c) Geometric mean IgG titers (GMT) as measured by His-capture ELISA to the wt autologous trimer immunogen following each inoculation. Immunizations are indicated by the vertical dashed gray lines. Six data points per time point per group were determined. Two independent ELISA experiments were performed and a representative experiment is shown.

To assess if N-glycan-deletion at the CD4bs altered the elicited B cell response and serum antibodies compared to unmodified trimers following vaccination, we performed an immunogenicity experiment in rabbits. We tested two different immunization regimens that involved priming animals with N-glycan-deleted trimers. We then compared each of these regimens to the control immunization regimen, where all animals were immunized with fully glycosylated trimers (Group 1). The rabbits from this control Group 1 were immunized four times with the parental trimer 16055 NFL TD CC (T569G), to which the N332 glycan had been introduced as described above (Fig 6a). For simplicity of the nomenclature, we will refer to this trimer as the “wt” control immunogen for the remainder of the study. The rabbits in Group 2 were immunized twice with the N-glycan deleted *+N332* N276Q/N360Q/N463Q/N301Q trimer (from now on, referred to as “ΔGly4”) and boosted two times with the wt immunogen (Fig 6a). The rabbits in Group 3 were immunized sequentially with the three N-glycan-deleted trimer variants: ΔGly4, then ΔGly2 (*+N332*N276Q/N463Q), then ΔGly1 (*+N332* N276Q) and lastly with wt trimer (Fig 6a) [52]. To enhance immune responses, we arrayed all trimers on liposomes at high-density as previously described [52]. We have demonstrated that this multivalent presentation of trimers on the surface of liposomes more effectively generates germinal centers B cells and serum neutralizing antibodies [52,53]. Animals from each group were immunized via the subcutaneous route at weeks 0, 4, 12 and 24 with 30 μg of each trimer arrayed on the liposomes (Fig 6b) and formulated in ISCOMATRIX adjuvant (CSL). We confirmed the quality of each trimer-liposome preparation by EM negative stain analysis prior to each immunization (Fig 6b).

**Fig 7:**
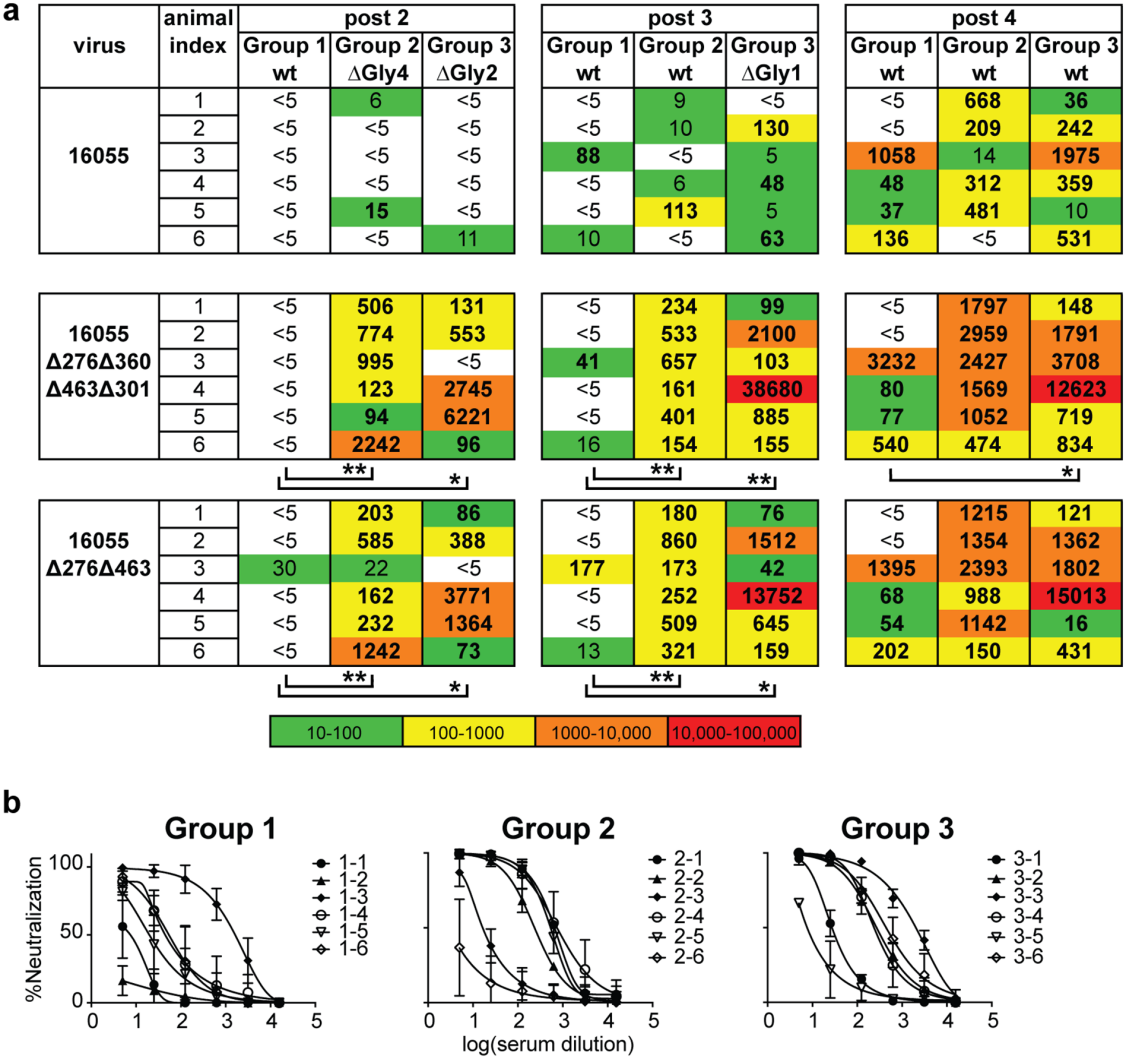
Neutralizing ID_50_ titers (reciprocal serum, fold-dilution) against 16055 N-glycan-deleted viruses. ID_50_ values are indicated in bold. Those derived by extrapolation are shown in non-bolded text (a) ID_50_ values for the viruses with the same N-glycan deletions proximal to the CD4bs as those in the trimer immunogens. Statistical differences were evaluated by the non-parametric Mann-Whitney test and, when detected at a level of significance, are indicated under the specific data set with * P<0.05 and ** P<0.01. (b) Serum neutralization curves for 16055wt virus derived from mean values for each data point of three independent TZM-bl-based neutralization assays. Error bars represent the standard deviation of the values from three independently performed experiments.

Bleeds were obtained on the day of immunization and 2 weeks after each immunization, except following the first inoculation (Fig 6a). After completion of the full regimen, we tested serum IgG binding titers against the *+N332* PT trimer by anti-His capture ELISA (See Methods and Fig 6c). There was no statistical difference in geometric mean binding titers (GMT) between Group 2 or Group 3 compared to Group 1, although the values obtained for the rabbits in Groups 2 and 3 displayed less variance following the fourth immunization (Fig 6c). We then analyzed the antibody neutralizing response of all animals in a longitudinal manner following the second, third and fourth immunization (post 2, post 3 and post 4, respectively). In terms of neutralizing capacity, the most striking difference for either Group 2 or Group 3 compared to Group 1 was observed with the N-glycan-deleted viruses. Specifically, we first analyzed the serum neutralizing capacity against the pseudoviruses with matching N-glycan deletions relative to the trimeric immunogens for Groups 2 and 3. Following two inoculations, all animals from Group 2 could neutralize the 16055Δ276Δ360Δ463Δ301 and the 16055Δ276Δ463 pseudoviruses and five of six animals from Group 3 neutralized these viruses. In contrast, only one animal in Group 1 weakly neutralized the 16055Δ276Δ463 virus after two immunizations. These differences were statistically significant (Fig 7a). The differences in neutralization capacity of the 16055Δ276Δ360Δ463Δ301 and 16055Δ276Δ463 pseudoviruses between Groups 2 or 3 compared to Group 1 were also significant following the third immunization. After the fourth immunization, when the animals from Groups 2 and 3 were both inoculated with the fully glycosylated wt trimers, there was a trend to higher titers against 16055Δ276Δ360Δ463Δ301 and 16055Δ276Δ463 viruses for Group 2 compared to Group 1. The difference for Group 3 in comparison to Group 1 for the four-N-glycan deleted (16055Δ276Δ360Δ463Δ301) virus was statistically significant (Fig 7a).

**Fig 8:**
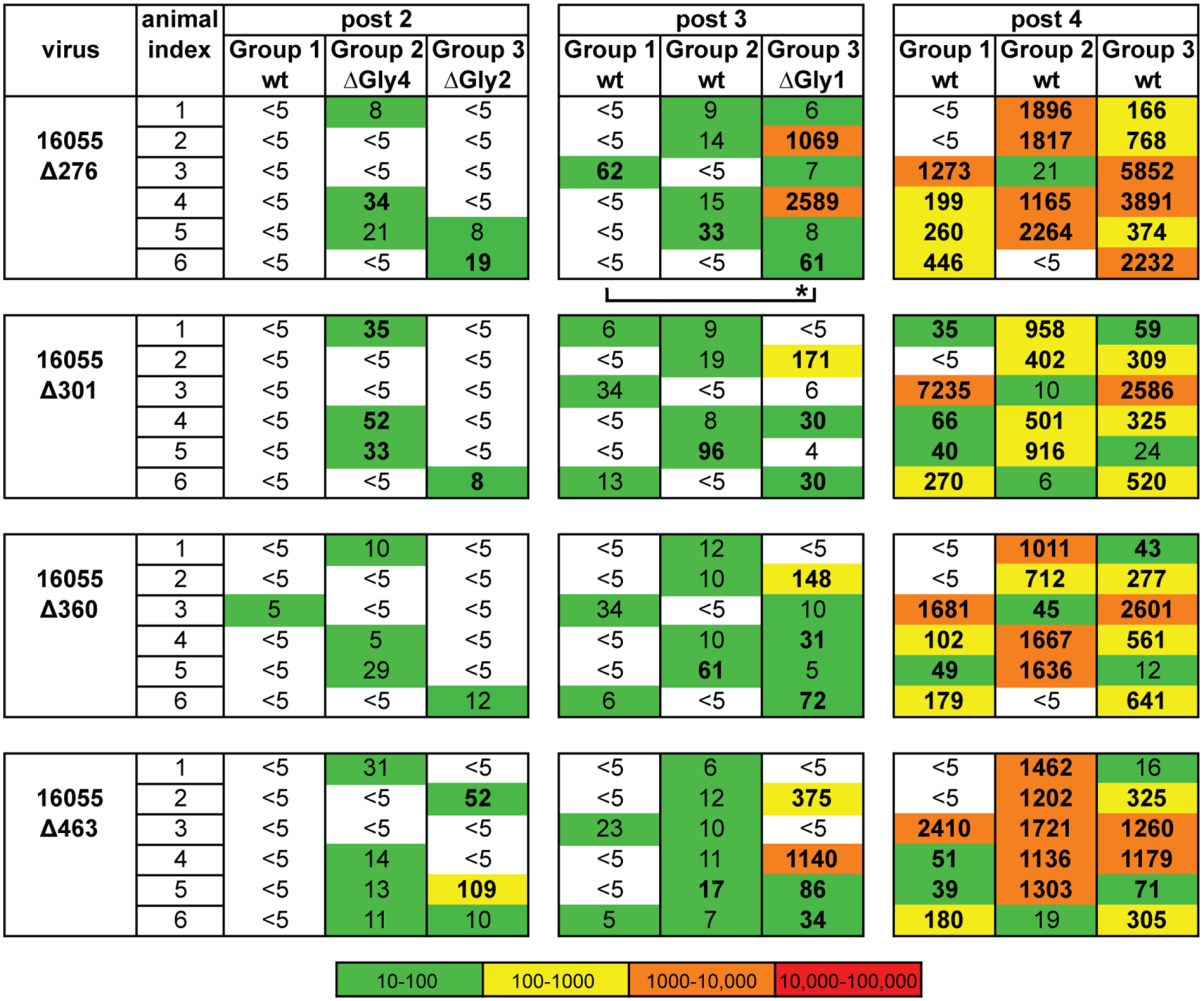
Neutralizing ID_50_ values for the singly N-glycan-deleted viruses. ID_50_ values are indicated in bold; those derived by extrapolation are shown in non-bolded text. Statistical differences were evaluated by Mann-Whitney test and, when detected, were indicated under each data set with * P<0.05.

Because the pseudoviruses with multiple glycan deletions were better neutralized by the serum derived from Group 2 or 3 animals compared to those from Group 1, we assessed neutralization against each of the 16055 singly-N-glycan-deleted virus Δ276, Δ360, Δ463 and Δ301 to define clearly the neutralization specificity in the polyclonal serum. That is, we sought to pinpoint if the elimination of single N-glycan would reflect the neutralization capacity detected against the multiple N-glycan deleted viruses (Fig 8). Several animals from Group 2 or Group 3 elicited weak, but detectable, neutralizing activity against all four of the single N-glycan-deleted viruses after the second immunization (week 4/post 2), while only the highest responder in Group 1, showed weak neutralization against 16055Δ360 at that time point (Fig 8). More animals in Group 2 or 3, compared to Group 1, exhibited neutralization serum activity against the singly glycan-deleted viruses after the third immunization (week 12/post 3). There was a statistically significant difference in titers between Groups 1 and 3 against the 16055Δ276 pseudovirus. After the fourth immunization (week 24/post 4), the neutralization titers against single glycan-deleted 16055 pseudoviruses increased substantially in all three groups although the tendency to display higher titers against single glycan-deleted viruses in either Groups 2 or 3, in comparison with Group 1, remained.

In terms of the specific viruses, titers against the 16055Δ301 pseudovirus did not increase more than two-fold in comparison with the titers against 16055wt, indicating that this N-glycan had a minimal effect in regards to neutralizing activity (Figs 7 and 8). In terms of specific animals from Group 2, the 16055Δ463 pseudovirus was better neutralized by the rabbit #2–3 (that is, animal number 3, from Group 2). This might be due to the peripheral location of the N463 glycan relative to the CD4bs providing better accessibility to the underlying protein surface (Fig 1a). Animals from Group 3 displayed high titers against the 16055Δ276 pseudovirus, and the difference in the responses between Group 1 and 3 was statistically significant after three immunizations. There was also a strong trend of more potent neutralization of the 16055Δ463 virus in this group after three immunizations, while the neutralization titer pattern for other single N-glycan-deleted viruses (16055Δ301 and 16055Δ360) was similar to the wt virus neutralization pattern at this time point. These results were consistent with a neutralizing antibody response focused toward the proximity of residue N276 by the ΔGly4, ΔGly2 and ΔGly1 sequential immunization, while responses proximal to residues 301 and 360 diminished, likely due to restoration of these N-glycans in the immunogens.

The trend of more potent and consistent neutralization elicited by the N-glycan deleted viruses was also detected when assessed against the autologous tier 2 fully-glycosylated 16055wt virus. The differences in 16055wt pseudovirus neutralization were detectable as well following the third immunization (post 3, Fig 7a). Four animals from Group 2 and five animals from Group 3 displayed neutralizing activity against the 16055wt, compared to only two animals from Group 1. After the final boost (post 4), five animals from Group 2 and six animals from Group 3 showed neutralization against 16055wt virus (Fig 7a). In terms of potency, four animals from each of these groups displayed autologous serum titers above 100, while only two animals displayed titers above 100 in Group 1 (Fig 7a). In general, the responses in the animals from Group 1 were less potent than those in either Groups 2 or 3, with only one animal achieving 100% neutralization against the wt autologous virus after four immunizations (Fig 7b), whereas, four animals in either Groups 2 or 3 achieved 100% wt virus neutralization (Fig 7b).

These data suggest that genetic deletion of PNGS proximal to the CD4bs on the Env trimeric immunogens may eliminate steric barriers imposed by the presence of N-glycans that normally limit the B cells responding to this conserved epitope. In our study, the elimination of these barriers led to a more consistent and robust neutralizing antibody response when the N-glycan-deleted immunogens were used to prime the immune response.

### A fraction of the neutralizing antibody response effectively targets the CD4bs

The analyses described in the previous section indicated that the neutralizing antibody responses were directed proximal to the CD4bs, especially in the sequential N-glycan-restored Group 3 animals. To determine by another means if the elicited neutralizing antibody response was in part directed to the CD4bs, we generated a pair of 16055gp120-based TriMut probes as previously described for the HXBc2 TriMut proteins [54]. Both 16055 gp120 variants possess three mutations (I423M, N425K, and G431E) in the bridging sheet (hence, TriMut) that allow recognition by CD4bs-directed antibodies, but eliminates binding to the primary HIV receptor, CD4 (S8 Fig). These modification permit the addition of the TriMut gp120 glycoproteins directly into the neutralization assays (“dump-in”) without affecting entry by the normal high-affinity binding of wt gp120 to CD4 [55,56]. The gp120 TriMut possesses an unmodified CD4bs, while the paired probe incorporates two additional mutations, D368R/M474A, which prevent binding by most CD4bs-directed antibodies (S8 Fig). These two isogenic proteins can be used to determine neutralization specificity directed toward the CD4bs by differential adsorption or depletion. We first validated the differential depletion assay using known bNAbs that can neutralize 16055, detecting a decrease in VRC13 and HJ16 neutralization upon the addition of the TriMut gp120, but not the isogenic 368R/474A variant (S9a Fig). The differential between the two proteins confirmed their capacity to map neutralization specific for the CD4bs (S9b Fig).

We then analyzed total polyclonal IgG isolated from selected hyperimmune rabbit anti-sera using this assay. Following IgG isolation, we established the concentration for each sample that could neutralize 80% of virus entry. Using this concentration of IgG, we then performed the adsorption assay. We determined that increasing amounts of the TriMut gp120 could deplete neutralizing activity of the wt 16055 virus, while the 368R/474A TriMut gp120 depleted only a portion of this activity (Table 1, S9 Fig). This differential indicated that some of the 16055-neutralizing activity was CD4bs-directed (Table 1, S9b Fig). We quantitated this differential neutralization at the CD4bs as a difference between TM and TM368R/474A area under the curve (AUC) values, normalized by the control AUC value (Table 1). We observed CD4bs-directed activity in rabbit #1–3, the highest responder from Group 1, after third and fourth immunizations (termed “post 3 and 4”; Table 1, S9c Fig). Rabbit #1–6 from Group 1 also showed partial CD4bs-directed neutralization activity. Rabbit #2–1 from Group 2 displayed a small fraction of neutralization directed to the CD4bs after the fourth immunization (Table 1, S9d Fig), while more than 50% of the total IgG neutralization in rabbit # 2–4 was directed against the CD4bs at this time point (Table 1, S9d Fig). In Group 3, however, two rabbits (#3–2 and #3–4) demonstrated partial CD4bs-directed neutralization following just the third immunization (post 3, S9e Fig, Table 1). Rabbit #3–4 displayed partial CD4bs-directed neutralization after fourth immunization, as well, whereas for rabbit #3–2 the CD4bs-directed differential was no longer detectable at this time point. In addition, following the fourth inoculation, three other rabbits from Group 3 displayed partial CD4bs-directed neutralizing activity (Table 1, S9e Fig).

**Table 1:** Quantification analysis of the neutralization mapping assay.

	time point	animal number	AUC Medium	AUC TriMut	AUC TriMut 368/474	TriMut adsorption, %	TriMut 368/474 adsorption, %	CD4bs differential
**Group 1**	post 3	1–3	8422	1741	3892	79	54	**32**
	post 4	1–3	7795	1205	3193	85	59	**30**
		1–4	7885	8131	7700	NA	NA	**NA**
		1–5	8083	8209	8167	NA	NA	**NA**
		1–6	8171	1345	3115	84	62	**26**
**Group 2**	post 3	2–5*	7901	1324	985.1	83	88	**0**
	post 4	2–1	7536	1062	1589	86	79	**8**
		2–2	6672	884.5	826.3	87	88	**0**
		2–4*	8193	1992	5423	76	34	**55**
		2–5*	8018	996.3	996.3	88	88	**0**
**Group 3**	post 3	3–2	8833	6211	6951	30	21	**28**
		3–4	8263	4240	7365	49	11	**78**
	post 4	3–1	8609	3118	3611	64	58	**9**
		3–2	8329	2106	1074	75	87	**0**
		3–3*	8193	2338	3955	71	52	**28**
		3–4	7993	2047	3853	74	52	**30**
		3–6*	8355	3240	4468	61	47	**24**
**Control Abs**		VRC13	9699	5995	9681	38	0	**100**
		HJ16	9942	5046	9761	49	2	**96**
		PGT145	8751	8760	8727	0	0	**0**

AUC—area under the curve. We calculated AUC for TriMut, TriMut 368/474 and the control curves. We calculated TriMut or TriMut 368/474 adsorption using equation $\frac{AUC\left(Medium\right)-AUC\left(TriMut\right)}{AUC\left(Medium\right)}\times 100\%$. We calculated CD4bs differential using equation $\frac{TriMut\;adsirption-TriMut368/474\;adsirption}{TriMut\;adsirption}\times 100\%$, so it is normalized by the total neutralization for each sample. Animals that showed heterologous cross neutralization are marked with *.

In sum, we observed CD4bs-directed activity in several animals from all three groups. Compared to animals from Group 1, animals from Group 3 showed more consistent CD4bs-directed neutralizing antibody responses following four immunizations.

### Purified serum IgG isolation and analysis reveals cross-neutralization

With indications that there was some CD4bs-directed neutralizing activity proximal to the CD4bs (and the proximal N-glycan at residue), we performed neutralization assays using the purified polyclonal IgG purified from serum of the rabbits that demonstrated weak serum neutralization against a selected panel of heterologous viruses. We analyzed neutralization of a small set of pseudoviruses with PNGS N276 deleted, namely BG505Δ276, JRFLΔ276, IAVIC22Δ276, along with their respective wt pseudoviruses. We also analyzed IgG neutralization of several pseudoviruses naturally lacking the N276 PNGS, Q259 and 62357, another Indian clade C pseudovirus from the same cohort as 16055, 1428, and the pseudoviruses 1086 and CE1176. We used the SIV pseudovirus as a negative control for neutralizing specificity as this virus is not recognized or neutralized by HIV Env-specific antibodies. For these experiments, we titrated the purified IgG starting at a relatively high initial concentration of 2 mg/ml because even in a hyper-immunized animal only a minor fraction of circulating IgG is antigen-specific (~5–10%), and, of that, only a subset is neutralizing. As a negative control, we used purified IgG isolated from a rabbit that was immunized similarly with blank liposomes in adjuvant, at the same concentrations, to rule out non-specific IgG effects in the cross-neutralization assay.

**Fig 9:**
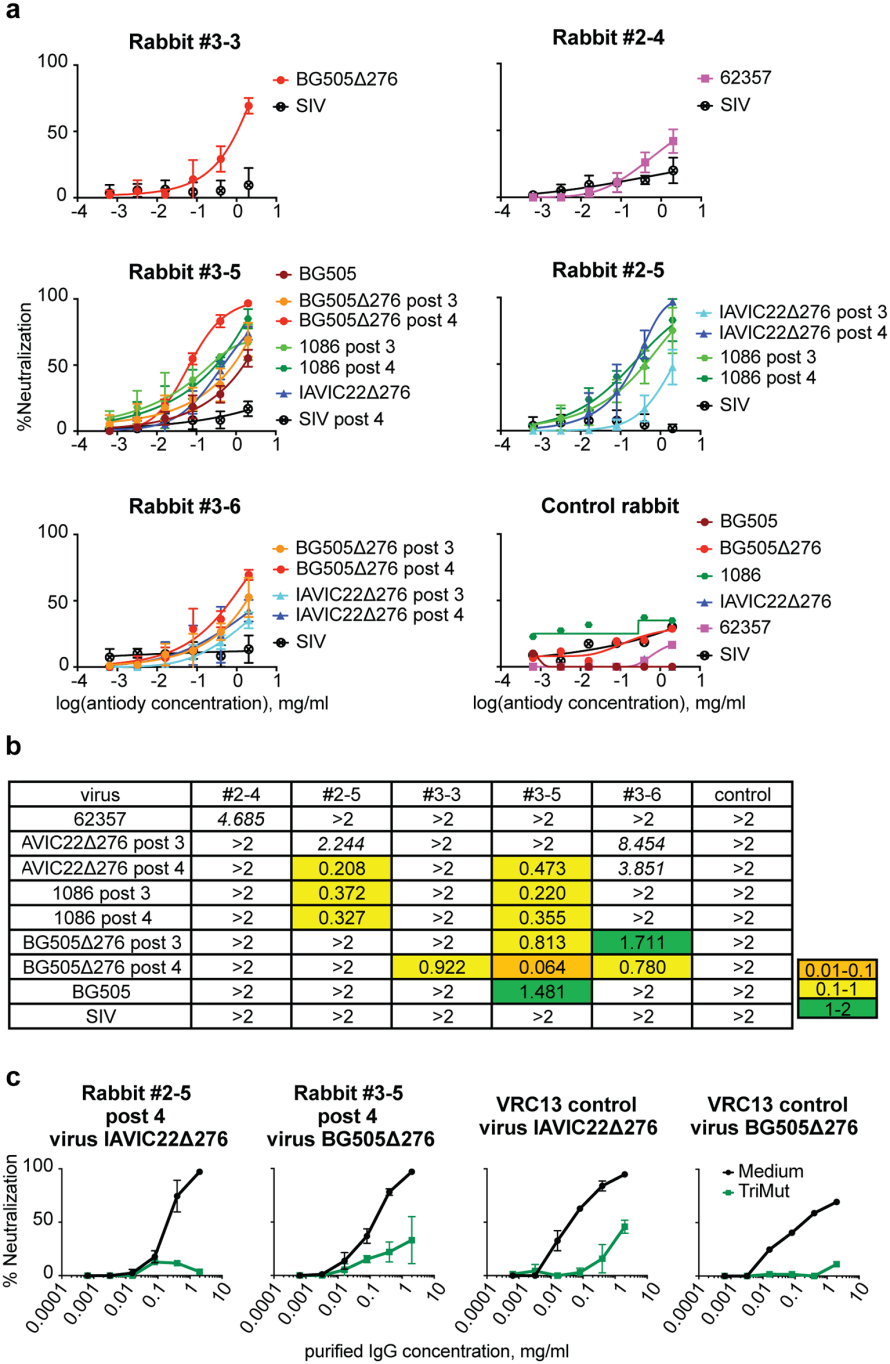
Purified serum IgG cross-neutralization. (a) IgG neutralization curves derived from mean values for each data point of three independent TZM-bl-based neutralization assays. Error bars represent the standard deviation. The rabbits are designated by the Group number first (1, 2 or 3) followed by a dash and the animal index number as indicated in Fig 7 (i.e., #3–5). If specified otherwise, the serum was analyzed following the fourth immunization. The “control rabbit” was immunized four times with blank liposomes in adjuvant and IgG was purified similarly to the experimental rabbit IgGs; the mean values of two experimental replicates are shown for this negative specificity control (b) ID_50_ values were derived from the curves described above and are color-coded as indicated. Weak neutralizing values were extrapolated based on the two highest IgG dilution data points and are indicated in italics. (c) Cross-neutralization of IAVIC22Δ276 and BG505Δ276 viruses analyzed by depletion with the 16055 gp120 TriMut protein. Purified IgG from the serum of rabbit #2–5 and rabbit #3–5 were titrated at the concentrations indicated (horizontal axis) in the absence or presence of the 16055 gp120 TriMut (two left panels). The 16055 gp120 TriMut protein was used at fixed concentration of 100 mg/ml. The mean values of two independent TZM-bl-based neutralization assays are shown with the bars at each dilution indicating the individual values. VRC13 IgG was used as a CD4bs-directed antibody positive control (two right panels) and in case of BG505Δ276 virus representative control experiment is shown.

We were able to detect weak cross-neutralization activity exclusively in IgG derived from animals in Group 2 or 3 that had been immunized with different variants of the N-glycan-deleted trimers (Fig 9). Most cross-neutralization was detected in the IgG isolated from the animals in Group 3 with three animals displaying detectable activity. Rabbit #3–3 displayed neutralization of the BG505Δ276 pseudovirus (Fig 9a and 9b), while rabbits #3–5 and #3–6 were able to neutralize both the BG505Δ276 and IAVIC22Δ276 pseudoviruses. In addition, rabbit #3–5 showed neutralization even against both the wt, fully glycosylated BG505 and 1086 pseudoviruses (Fig 9a and 9b). Following three immunizations, rabbit #3–6 neutralized BG505Δ276 and this activity increased following four inoculations. Two animals from Group 2 displayed some detectable cross-neutralizing activity. Rabbit #2–5 was able to neutralize the IAVIC22Δ276 and 1086 pseudoviruses following three immunizations and this activity increased against the IAVIC22Δ276 pseudovirus following the fourth immunization (Fig 9a and 9b). Rabbit #2–4 very weakly neutralized the 62357 (NIH15) pseudovirus after the fourth immunization (Fig 9a and 9b), which naturally lacks the N-glycan at residue 276. None of the IgGs derived from the Env-trimer-immunized cross-neutralized the control SIV pseudovirus, confirming HIV cross-neutralization specificity. Finally, even though we observed some CD4bs-directed neutralization in two animals from Group 1 in the previously described depletion assay, we were not able to detect cross-neutralizing serum activity in any IgG isolated from animals in this group.

To further confirm specificity of the cross-neutralization, we performed a depletion assay with the 16055 gp120 TriMut probe for the animals displaying the highest IgG IC_50_ values, i.e. rabbit #2–5 for the IAVIC22Δ276 pseudovirus and rabbit #3–5 for the BG505Δ276 pseudovirus (Fig 9c). We demonstrated that the cross-neutralizing activity was adsorbed substantially by pre-incubation of IgG with the 16055 gp120 TriMut protein, indicating that, in those animals, this activity was HIV Env-specific.

Overall, cross-neutralization was consistent with the CD4bs mapping for the animals from Group 2 and Group 3, thus, most animals with CD4bs-directed IgG neutralizing activity showed some level of cross-neutralization (marked with * in Table 1), except one rabbit from Group 3 (#3–5). This animal displayed generally low autologous neutralization and therefore the response could not be analyzed in the mapping experiment. Together, these data suggest that the sequential ΔGly4 to ΔGly2 to ΔGly1 immunization did better than the other two regimens at directing the neutralizing antibody response to the CD4bs.

## Discussion

Coupled with quaternary packing, N-linked glycosylation prevents most naïve B cells from gaining a “foothold” against the underlying Env trimer polypeptide surface to prime neutralizing Ab responses. Accordingly, in this study, we generated well-ordered and highly stable 16055 NFL trimers possessing targeted PNGS deletions proximal to the CD4bs to better expose this conserved neutralizing determinant for BCR access and B cell activation. We demonstrated that up to four specific PNGS can be deleted without altering trimer conformational integrity as determined by SEC, DSC, EM and by efficient recognition by selected trimer-specific bNAbs. We further showed that these same PNGS can be deleted in the context of full-length Env to generate pseudoviruses that maintain a tier 2-like phenotype as determined by selected antibodies and HIVIG. In a rabbit immunogenicity study, we demonstrated that PNGS-deleted 16055-stabilized NFL trimers more efficiently prime neutralizing antibody responses, and that there was a statistically significant difference in the capacity to neutralize the glycan-deleted pseudoviruses between the regimens that incorporated PNGS-deleted trimers and the control regimen that incorporated only wt trimers. We also detected a tendency to have more potent neutralization against the tier 2 autologous 16055wt pseudovirus in the animals immunized with the PNGS-deleted trimer variants. In addition, even though we only used a single Env strain in our immunogenicity experiment, we observed some cross-neutralization activity in several immunized animals from both groups “primed” with the CD4bs N-glycan-deleted trimers, notably in some animals that were sequentially boosted with the PNGS-restored immunogens.

We initially visited the approach to delete PNGS proximal to the CD4bs in the context of gp120 [50]. Here, we generated PNGS deletions in the context of well-ordered trimers, to eliminate steric barriers for antibody recognition imposed by the N-glycans surrounding the CD4bs, while maintaining the steric trimer constraints imparted by the trimeric nature of our trimeric Env. The fact that the same PNGS that we eliminated in our NFL immunogens can be altered in the context of native 16055 Env when pseudo-typed as viruses to mediate functional entry is reassuring concordance between the NFL trimer design and native Env. N-glycan deletions that were not compatible with native trimer formation were often highly conserved PNGS that were previously shown to be critical for folding of gp120 itself [57]. The fact that the 16055 NFL TD CC (T569G) trimers can tolerate the described N-glycan deletions attest to their stable design [33,34]. In the 16055 NFL trimers described here, deletion of the PNGS at N197 was detrimental, in contrast with the results recently described deletion of this N-glycan in the BG505 SOSIP context [35,58]. Deletion of N-glycan 301 in the 16055 Env context does not make the virus more globally sensitive, in contrast with results reported previously for YU2 or JRFL or SS1196 pseudoviruses [50,51], which become sensitive to the non-broadly neutralizing mAbs, F105 and 447-52D upon removal of the N-glycan at residue 301.

The aim of the trimer redesign by targeted N-glycan deletion is to enhance B cell access to the CD4 binding loop and proximal elements to ultimately generate a cross-reactive antibody response when used as immunogens. One caveat to this approach is that removal of the N-glycan will expose the underlying protein surface, potentially rendering it immunogenic. We would envision that although some of the immune response will be elicited to epitopes that will no longer be accessible in the context of wt virus with a fully intact glycan shield, a fraction of the B cell response will be able to access the CD4bs. And that there is a general advantage of increasing B cell activation to the glycan-denuded region so that some of these responses can be driven to accommodate the shield by gradual restoration of N-glycans in either a homologous or heterologous context. In this regard, our immunogenicity results suggest that sequential restoration of N-glycans proximal to the CD4bs may help to focus the antibody response on either the available protein epitope free of glycans and/or to the precise CD4bs itself. This interpretation is consistent with the data as we observed a statistically significant difference in neutralization of 16055Δ276 pseudovirus from Group 3 samples following three immunizations compared with the control group, Group 1. More animals from the Group 3 demonstrated partial CD4bs-directed neutralization compared to animals from Group 1, again suggestive of B cell focusing at the conserved CD4bs. For a more definitive answer to this issue, isolation of individual CD4bs-directed B cells and cloning of monoclonal antibodies is needed. Importantly, we detected weak but specific cross-neutralization of selected heterologous viruses, mostly lacking N-glycan 276, which is known to be a major impediment toward potent vaccine-elicited neutralization at the CD4bs, even in gL-reverted transgenic mice [59]. We detected weak neutralization of wt BG505 pseudovirus in one rabbit from Group 3 suggesting that this impediment can be overcome. Strategies to boost these heterologous responses are needed to increase the robustness of this approach.

Other investigators have explored the effect of glycan-shield disruption at the CD4bs on B cell activation and germline reverted antibodies binding enhancement in vitro or in germline transgenic or chimeric mice [59–61]. In some cases, the stimulation of germline reverted BCRs in vitro and in vivo was observed [60,61]; however, with limited autologous neutralization [59]. Two recent studies performed in parallel to ours used similar glycan-deleted immunogens [35,36] in outbred animals, but without the boosting regimens we described here. Crooks et al. used JRFL Env based trimer VLPs both possessing (wt) and lacking the N-glycan at N362. They detected some autologous neutralization and mapping to CD4bs-proximal N-glycans [36], but with small numbers of animals per group it was not possible to determine statistical difference in the responses against wt or N362 glycan-deleted JRFL virus [36,62]. Zhou et al. analyzed four well-ordered SOSIP trimers possessing targeted N-glycan deletions at the CD4bs including those derived from 16055-based chimeric trimer. Homologous 16055 wt virus neutralization was observed in two out of four 16055–2.3-chim.DS.SOSIP.ΔGly4–immunized animals after three immunizations, where their “ΔGly4” included N197, N463 and N276 PNGSs modifications with N362 naturally missing [35]. Differences in 16055 autologous neutralization responses might be attributed to our use of trimers arrayed on liposomes and slightly different N-glycan deletions between the two immunogens. This study also detected some cross-neutralization of N-glycan deleted pseudoviruses, consistent with the results presented here. Note that there are substantial differences between these studies such as our regimen used trimer-liposomal array, included the gradual restoration of the deleted N-glycans and we used more animals per group to allow better statistical analysis. In addition, our regimen consisted of four immunizations, and a long interval between the third and fourth immunizations, which we have shown previously enhances neutralizing antibody responses [63].

In sum, the targeted N-glycan approach outlined in this study shows promise to focus B cell responses to the CD4bs. Targeted N-glycan deletion may be applicable to other neutralizing determinants present on this extensively glycan-shrouded critical protein complex, thereby allowing recognition and engagement of naïve B cells that otherwise would not be efficiently activated by the fully glycosylated trimeric complex.

## Methods

### Site-directed mutagenesis

The described Env DNA substitutions were introduced via site-directed mutagenesis PCR using a QuikChange Lightning Multi Site-Directed Mutagenesis kit (Agilent Technologies) into NFL expressing plasmids (CMV-R, where CMV is cytomegalovirus) [34] or into the pcDNA plasmid, containing codon-optimized 16055 *env* sequences. In brief, single primers were designed for each mutation. We used up to three primers per reaction mixture to introduce multiple substitutions simultaneously. Reaction products were transformed into competent bacteria and plated onto Luria broth agar plates for colony selection, subsequent plasmid DNA isolation, and sequencing. To map serum neutralizing activity directed toward the CD4bs, TriMut and TriMut 368R/474A proteins were generated as described previously [54]. Briefly, three mutations, I423M, N425K and G431E, were introduced to make a triple mutant 16055 gp120 protein (TriMut) that eliminates CD4 binding but does not affect recognition by CD4bs-directed mAbs. For the receptor-binding-defective protein, TriMut 368R/474A, two additional mutations, D368R and M474A, were introduced to eliminate CD4 binding.

### Expression and purification of HIV Env

The Env NFL trimeric proteins and TriMut proteins were produced as previously described [38,64]. Briefly, the 16055 Env proteins were transiently expressed as soluble glycoproteins in 293F (Free-style 293-F Cells, Thermo Fisher Scientific) cells from codon-optimized sequences under the control of the CMV promoter/enhancer [34]. Cell culture supernatants were harvested at day 5 post-transfection, and the Env-derived glycoproteins were purified by affinity chromatography using a *Galanthus nivalis* lectin-agarose column (Vector Laboratories). Bound glycoproteins were eluted with phosphate buffered saline (PBS) containing 500 mM NaCl and 500 mM methyl-α-D-mannopyranoside and then concentrated with an Amicon filter (30-kDa) to 1 ml. The lectin-purified proteins were subsequently purified by size-exclusion chromatography (SEC) using a HiLoad Superdex 200 16/60 column to separate the trimer and gp120 monomer fractions.

### Differential scanning calorimetry (DSC) studies

Thermal stability of the soluble 16055 trimer and its N-glycan-deleted variants were evaluated using MicroCal VP-Capillary differential scanning calorimetry instrument (General Electric). Protein samples were dialyzed in PBS, pH 7.4, and the concentrations were adjusted to 0.125 mg/ml. Scans were collected at a rate of 1°C per min over a temperature range of 20–100°C, while pressure was maintained at 3.0 atm throughout the scan period. DSC data were analyzed after buffer correction, normalization, and baseline subtraction using CpCalc software provided by the manufacturer.

### Electron microscopy (EM) sample preparation

The purified NFL trimers were analyzed by negative-stain electron microscopy (EM) following the same protocol previously described [34] Data were collected using an electron dose of ~30e^-^/Å^2^. All the data were processed as previously published [34]. Briefly, particles were picked and assembled into a stack using the Appion software package [65] Iterative multivariate statistical analysis (MSA)/multireference alignment (MRA)) was used to obtain 2D classes. Using EMAN2 [66] we obtained EM volumes of the trimers in complex with the VRC03 Fab. We used 2475 particles to obtain the 3D volume of the *+N332* PT in complex with 3 VRC03 Fabs and 3250 particles for the asymmetric volume bound to 2 VRC03. For the 3D reconstruction of the *+N332* N276Q/N360Q/N463Q/N301Q trimer bound to 3 VRC03 Fabs, 2448 particles were used.

### Enzyme-linked immunosorbent assay (ELISA)

His-capture ELISA was performed as previously described [34]. In brief, MaxiSorp plates (Thermo) were coated overnight at 4°C with 1.5 μg/ml of a mouse anti-His tag monoclonal antibody (mAb) (R&D Systems) in PBS, pH 7.5. The next day the plates were incubated at 4°C in blocking buffer (2% BSA in PBS, pH 7.5) for 2 h and the Env-derived soluble trimers was added to the plate at a concentration of 3 μg/ml in PBS and incubated at RT for 40 min. Serially diluted mAbs at a maximum concentration of 10 μg/ml or sera from vaccinated animals were added into wells, and following incubation and washing, the secondary antibodies of peroxidase-conjugated goat anti-human IgG or goat anti-rabbit IgG were added to all wells. Following incubation and washing, the signals were developed by addition of the 3,3’,5,5;-tetramethylbenzidine chromogenic substrate solution (Life Technologies) and detected at 450 nm. For direct-coat ELISA, trimers were added directly to the wells at 3 μg/ml and analyzed for antibody binding as described above.

### Bio-layer interferometry (BLI) binding analysis and kinetics

The kinetics of VRC03 Fab binding to glycan-deleted trimer varians were performed with an Octet RED96 system (ForteBio Inc, Menlo Park, CA) by BLI in a 96-well format. The trimers were subjected to SEC to remove undesired oligomeric forms where applicable. Then trimers were captured by anti-His biosensors (HIS2; ForteBio) at concentration 10 μg/ml and VRC03 Fab were used as analytes in solution (1000 nM–15.6 nM). Ab-Env associations (on-rate, Kon) were measured over a 2 min interval, followed by immersion of the sensors into wells containing buffer to measure dissociation (off-rate, K_dis_). KD values (in nanomolar units) were calculated as off-rate/on-rate (K_dis_/K_on_). The sensograms were corrected with the blank reference and fit with the software ForteBio Data Analysis 7 using a 1:1 binding model with the global fitting function (grouped by color, R_max_).

### Ethics statement

The rabbit immunogenicity study was performed at The Scripps Research Institute (TSRI), a site approved by the Association for Assessment and Accreditation of Laboratory Animal Care (AAALAC). The animal inoculation protocols were approved by TSRI’s Institutional Animal Care and Use Committee (IACUC). protocol #10–0002, which was designed and conducted in strict accordance with the recommendations of the NIH *Guide for the Care and Use of Laboratory Animals*, the Animal Welfare Act and under the principles of the 3Rs. All efforts were made to minimize discomfort related to the inoculations and blood collection.

### Animal immunization

For the immunogenicity experiment New Zealand White female rabbits (six per group) were immunized at weeks 0, 4, 12 and 24 with 30 μg of each trimer arrayed on the liposomes as described in [52]. Briefly liposomes were prepared using mixture of DSPC (1,2-distearoyl-sn-glycero-3-phosphocholine), cholesterol, DGS-NTA(Ni2) in molar ratio 60:36:4, respectively. The components were dissolved in chloroform, mixed and placed overnight in a desiccator under vacuum to yield a lipid film. The lipids were hydrated in PBS for 2 hr at 37°C, with constant shaking followed by vigorous sonication. The liposomes were extruded by sequentially passing across a series of membrane filters (Whatman Nuclepore Track-Etch membranes) with pore sizes of 1.0, 0.8, 0.2, and 0.1 m, respectively. The liposomes were incubated overnight with trimer proteins (900 μg protein to 300 μl liposomes) and passed over a S200 size-exclusion column to separate the protein-coupled liposomes from unbound protein. Quality of each trimer-liposome preparation was confirmed by EM negative stain analysis prior to each immunization. Trimer-coupled liposomes were formulated with 75 units of ISCOMATRIX adjuvant (CSL, Australia) and used for rabbits immunization via the subcutaneous route. Serum was collected on the day of inoculation and 2 weeks after each immunization to assess binding and neutralization titers.

### Neutralization assays

Standard TZM-bl-based neutralization assays were performed as previously described [67,68] using 16055 full-length Env natural sequence to complement the Env-deleted plasmid to generate clade C pseudovirus [69] and its deglycosylated variants. Titrated 16055 pseudovirus was used to evaluate sensitivity and inhibition of entry (neutralization, IC_50s_) to a panel of mAbs (VRC01, VC03, VRC06b, HJ16, F105, b6, GE136, 17b, PGT145, 447-52D, 19b) and HIV Immunoglobulin (HIVIG, lot# 140406), derived from a pool of HIV-infected individuals. Once characterized, the 16055 pseudoviruses were pre-incubated with serum samples derived from the vaccinated rabbits to determine anti-serum neutralization capacity. Neutralization titers were expressed as antibody concentrations sufficient to inhibit virus infection by 50% (EC_50_) or as the serum dilution factor sufficient to inhibit virus infection by 50% (ID_50_). Spearman’s Rank Correlation analysis of neutralizing titers and DSC-determined T_m_ was performed using Prism 6 software (GraphPad).

To examine the contribution of potential CD4bs-directed antibodies to the serum neutralizing activity, neutralization assays were performed using the isogenic TriMut and TriMut D368R/D474A 16055 gp120 pair as Env-specific antibody-adsorbing probes as described previously [54]. The D368R mutation eliminates gp120 (or trimer) binding to CD4 on the TZM-bl target cells in the neutralization assay so that the proteins can be added to serum for pre-incubation and then remain in the assay during assessment of viral entry. This assay is a modified version of the standard neutralization assay described above. To perform this analysis, we purified total IgG from the serum samples obtained after the third and fourth immunization, using 2 ml of serum and 600 μl of equal parts of Sepharose A and G (GE Healthcare Life Sciences) equilibrated in PBS. After overnight incubation at 4°C, we washed the resin with 15 ml of PBS and eluted with 4 ml of IgG elution buffer (Thermo Fisher Scientific). The eluates were neutralized with 400 μl of 1M Tris HCl pH 8.0 and dialyzed against PBS. Each serum IgG sample was titrated against 16055 virus in TZM-bl-based neutralization assay as described above. Before addition of pseudovirus, 100 μl of each total serum IgG sample at IC_80_ was pre-incubated with serial dilutions of TriMut, TriMut 368/474, or cell culture medium (12.5 μl), respectively, for 1 hour at 37°C. For each purified IgG, two neutralization assays were performed.

### Statistical analysis

We used the unpaired two-tailed Mann Whitney test when comparing neutralization values from Group 1 animals to samples derived from either Group 2 or Group 3 subjects. This nonparametric test that does not assume Gaussian distribution of values with 6 subjects per group.

## Supporting information

S1 Fig SEC profiles and EM 2D class averages of lectin affinity-purified glycan deleted trimers lacking the 332 N-glycan. Panels A1, A2 and A3 indicate trimers with one, two or three Group A PNGS-mutations, respectively. Panel B1 indicates trimers with one PNGS mutated from Group B. SEC profiles of mutated trimers (solid line) are shown in comparison with the PT (parental trimer, dotted line) and the expression level relative to expression level of PT is shown on each SEC graph. The percentage of native-like trimers determined by negative stain EM (the sum of closed and open native-like trimers) for each mutant trimer protein is indicated above the 2D class averages. Four single-particle representative images shown for each variant.
(TIF)

S2 Fig DSC thermal transition (T_m_) curves. The curves and derived T_m_s of glycan-deleted trimers (red solid line) compared to the backbone PT protein lacking N332 (black dotted line) are shown. Panels A1, A2 and A3 indicate trimers with one, two or three Group A PNGS-mutations, respectively. Panel B1 indicates trimers with one PNGS mutated from Group B.
(TIF)

S3 Fig Comparison of the 16055 NFL TD CC trimers without (PT) and with the 332 N-glycan (*+N332* PT). (a) DSC thermal transition curves and derived T_m_s of PT and *+N332* PT trimers. (b) EM 2D class averages. Percentage of native-like trimers determined by negative stain EM (the sum of closed and open native-like trimers) for each trimer is indicated above the 2D class averages; 16 representative single-particle images are shown for each variant. (c) ELISA binding curves of selected antibodies to the PT (blue) and *+N332* PT (red) proteins. His-captured trimers were analyzed. Experimental duplicates were analyzed for each antibody dilution, mean values are shown.
(TIF)

S4 Fig CD4bs-specific antibody binding profiles of the glycan deleted trimer. (a) Schematic presentation of N-glycan composition proximal to the trimer CD4bs in the selected glycan-deleted trimers. Filled blue triangle—the N-glycan is present; empty blue triangles—the N-glycan is genetically deleted or naturally absent (residue 332). (b) Comparison of the PT (dark blue) and N276Q/N463 (green) trimers. His-captured trimers were analyzed. Experimental duplicates were analyzed for each antibody dilution, mean values are shown.
(TIF)

S5 Fig Antibody binding profiles of the glycan-deleted trimers. (a) Comparison of the PT (dark blue) and N276Q/N463 (green) trimers. (b) (b) Comparison of the PT (dark blue) and N276Q/N463 (green) trimers. His-captured trimers were analyzed. (c) Comparison of the *+N332* PT (dark blue) with *+N332* N301Q (yellow), *+N332* N276Q/N360Q/N463 (red) and *+N332* N276Q/N360Q/N463/N301Q (light blue) trimers. His-captured trimers were analyzed. (d) 2G12 binding of the trimers coated directly on the ELISA plate. Experimental duplicates were analyzed for each antibody dilution, mean values are shown.
(TIF)

S6 Fig EC_50_ values of antibody binding to the N-glycan-deleted trimers. (TIF)

S7 Fig EM analysis of the trimer—VRC03 Fab complexes. **(a)** Reference free 2D classes of *+N332* PT in complex with VRC03 (left panel) and *+N332* N276Q/N360Q/N463Q/N301Q in complex with VRC03 (right panel). Red: 3 Fabs bound, orange: 2 Fabs bound, green: 1 Fab bound, and blue: unbound trimers. (b) Table listing the occupancy of VRC03 Fab relative to the trimers. (c) EM 3D reconstructions of *+N332* PT in complex with VRC03 (top panel; symmetry C3 applied) and *+N332* N276Q/N360Q/N463Q/N301Q in complex with VRC03 (lower panel; symmetry C3 applied). The crystal structure of the BG505 soluble trimer in complex with PGV04 (PDB:3J5M) was fitted inside the EM volumes. The contour levels used for the symmetric volumes (C3) were ~19.
(TIF)

S8 Fig Characterization of probes for the neutralization depletion assay. Based on 16055 gp120, two probes, TriMut with triple mutations (I423M, N425K and G431E) and TriMut 368/474 with two additional mutations (D368R and D474A), were designed to map the CD4bs neutralizing antibodies present in sera by neutralization depletion assay. To characterize the binding profile of the probes by Biolayer Interferometry (BLI), a panel of antibodies and CD4-Ig were captured by anti-human IgG Fc sensor and then dipped into 200 nM of probes in the well. The association and dissociation times are 3 min, respectively.
(TIF)

S9 Fig Neutralization adsorption assay with the 16055 gp120 TriMut and TriMut 368/474 probes. Serum samples with neutralization titers above 100 were used to isolate total IgGs. The purified IgG samples were used in the assay at IC_80_ concentration. (a) panel confirms the differential depletion capacity of TriMut and TriMut 368/474 probes with CD4bs specific VRC13 and HJ16 bNAbs. PGT145 was used as a negative control. (b) A graphical depiction of the CD4bs differential is shown. Differential assays for Group 1 (c), Group 2 (d) and Group 3 (e) are shown. Two independent adsorption experiments were performed and a representative experiment is shown.
(TIF)
