# Supplementary figures and images for: Targeted N-glycan deletion at the receptor-binding site retains HIV Env NFL trimer integrity and accelerates the elicited antibody response
Source: PLoS Pathog. 2017 Sep 13;13(9):e1006614. doi: 10.1371/journal.ppat.1006614 (PMC5640423; doi:10.1371/journal.ppat.1006614)

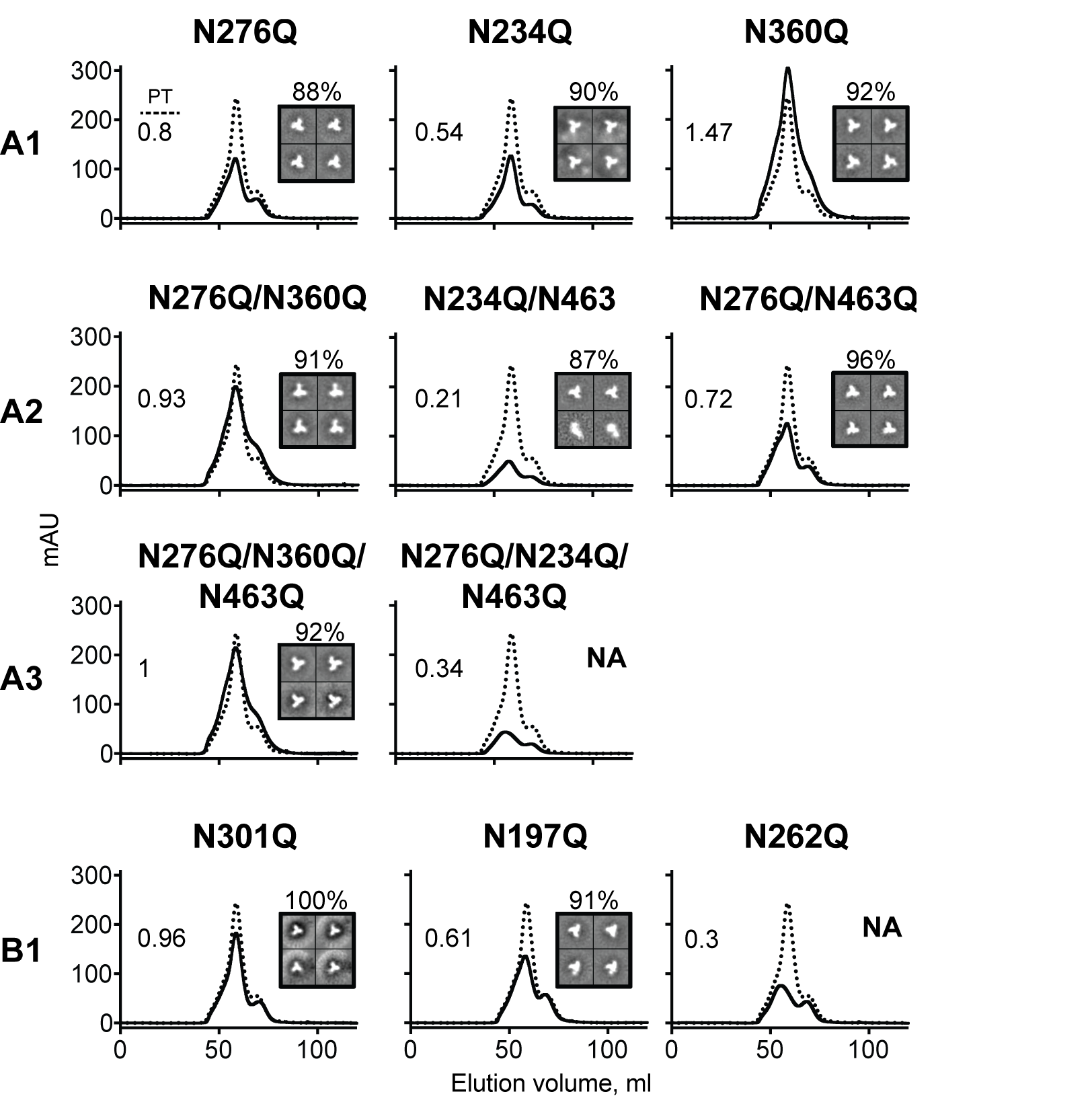

Supplement: S1 Fig — Panels A1, A2 and A3 indicate trimers with one, two or three Group A PNGS-mutations, respectively. Panel B1 indicates trimers with one PNGS mutated from Group B. SEC profiles of mutated trimers (solid line) are shown in comparison with the PT (parental trimer, dotted line) and the expression level relative to expression level of PT is shown on each SEC graph. The percentage of native-like trimers determined by negative stain EM (the sum of closed and open native-like trimers) for each mutant trimer protein is indicated above the 2D class averages. Four single-particle representative images shown for each variant. (TIF) [file ppat.1006614.s001.tif]

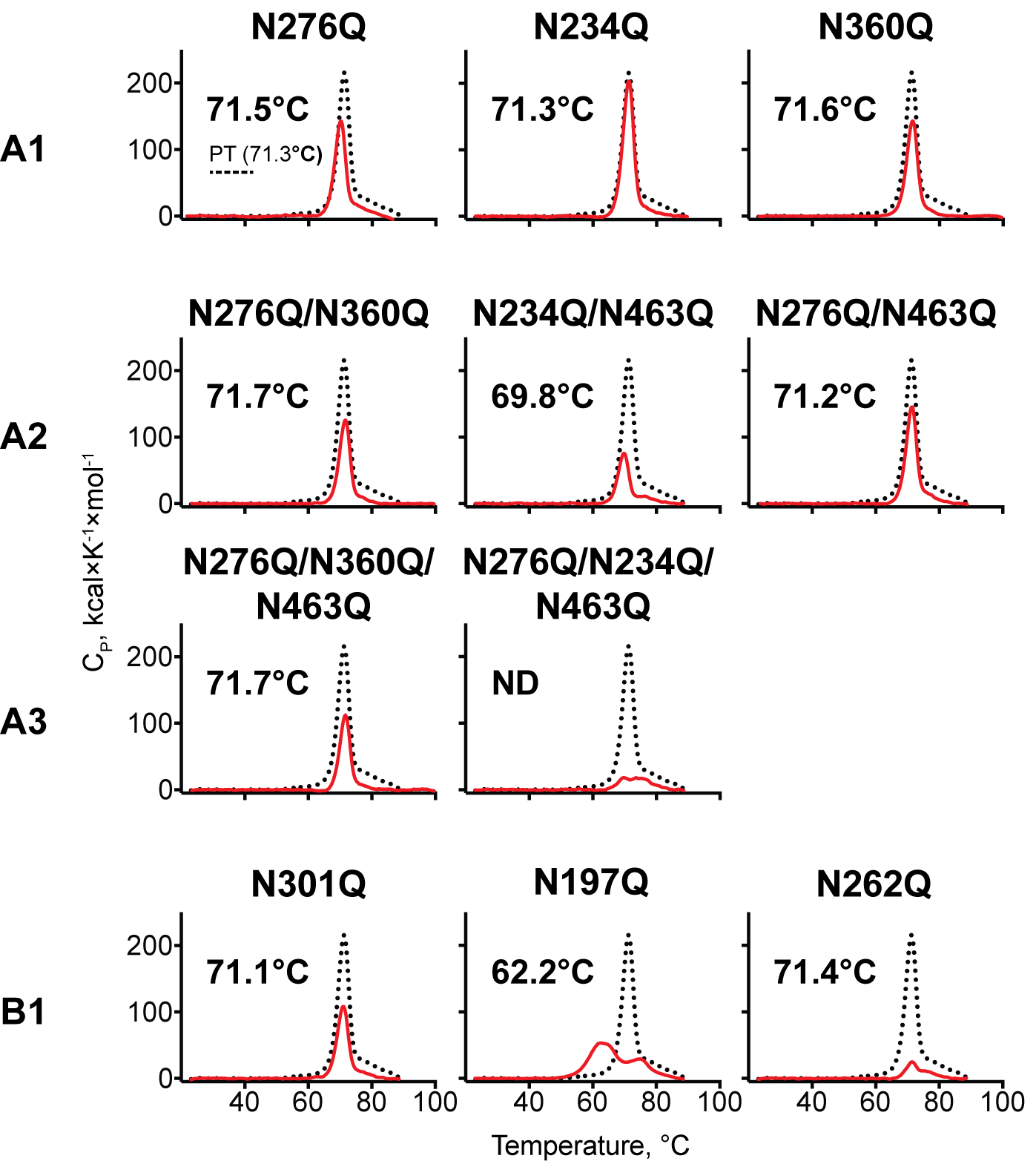

Supplement: S2 Fig — The curves and derived Tms of glycan-deleted trimers (red solid line) compared to the backbone PT protein lacking N332 (black dotted line) are shown. Panels A1, A2 and A3 indicate trimers with one, two or three Group A PNGS-mutations, respectively. Panel B1 indicates trimers with one PNGS mutated from Group B. (TIF) [file ppat.1006614.s002.tif]

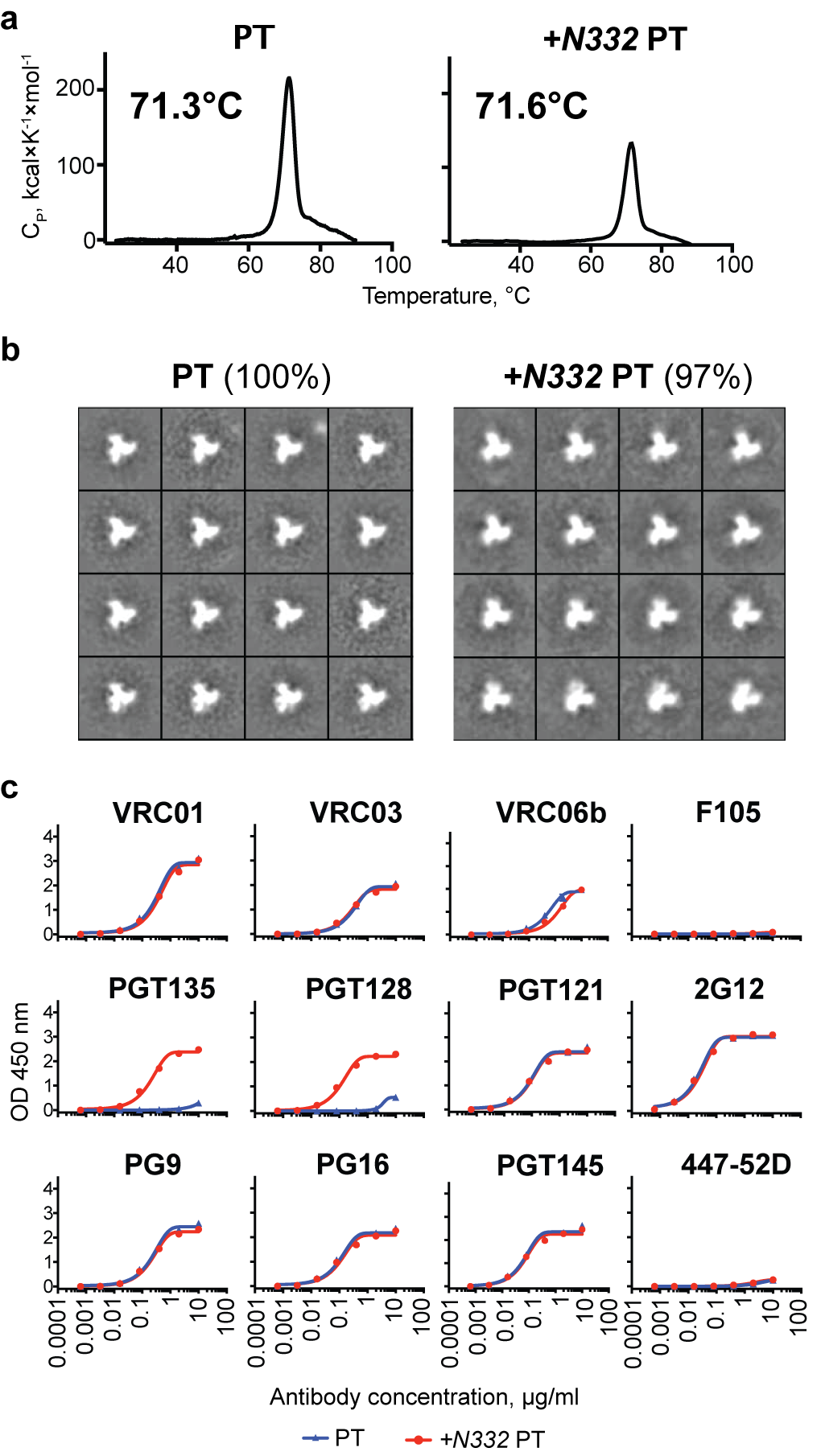

Supplement: S3 Fig — (a) DSC thermal transition curves and derived Tms of PT and +N332 PT trimers. (b) EM 2D class averages. Percentage of native-like trimers determined by negative stain EM (the sum of closed and open native-like trimers) for each trimer is indicated above the 2D class averages; 16 representative single-particle images are shown for each variant. (c) ELISA binding curves of selected antibodies to the PT (blue) and +N332 PT (red) proteins. His-captured trimers were analyzed. Experimental duplicates were analyzed for each antibody dilution, mean values are shown. (TIF) [file ppat.1006614.s003.tif]

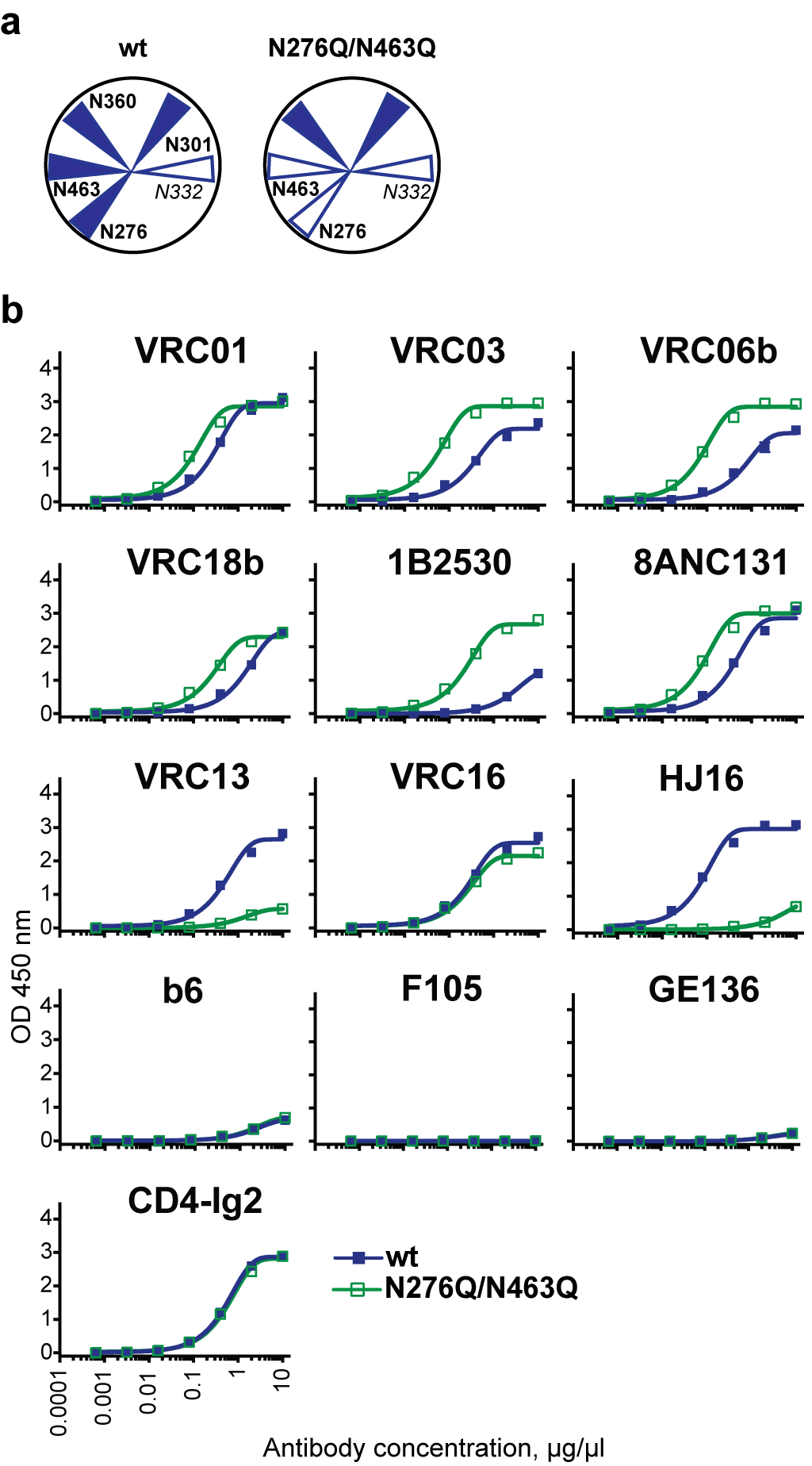

Supplement: S4 Fig — (a) Schematic presentation of N-glycan composition proximal to the trimer CD4bs in the selected glycan-deleted trimers. Filled blue triangle—the N-glycan is present; empty blue triangles—the N-glycan is genetically deleted or naturally absent (residue 332). (b) Comparison of the PT (dark blue) and N276Q/N463 (green) trimers. His-captured trimers were analyzed. Experimental duplicates were analyzed for each antibody dilution, mean values are shown. (TIF) [file ppat.1006614.s004.tif]

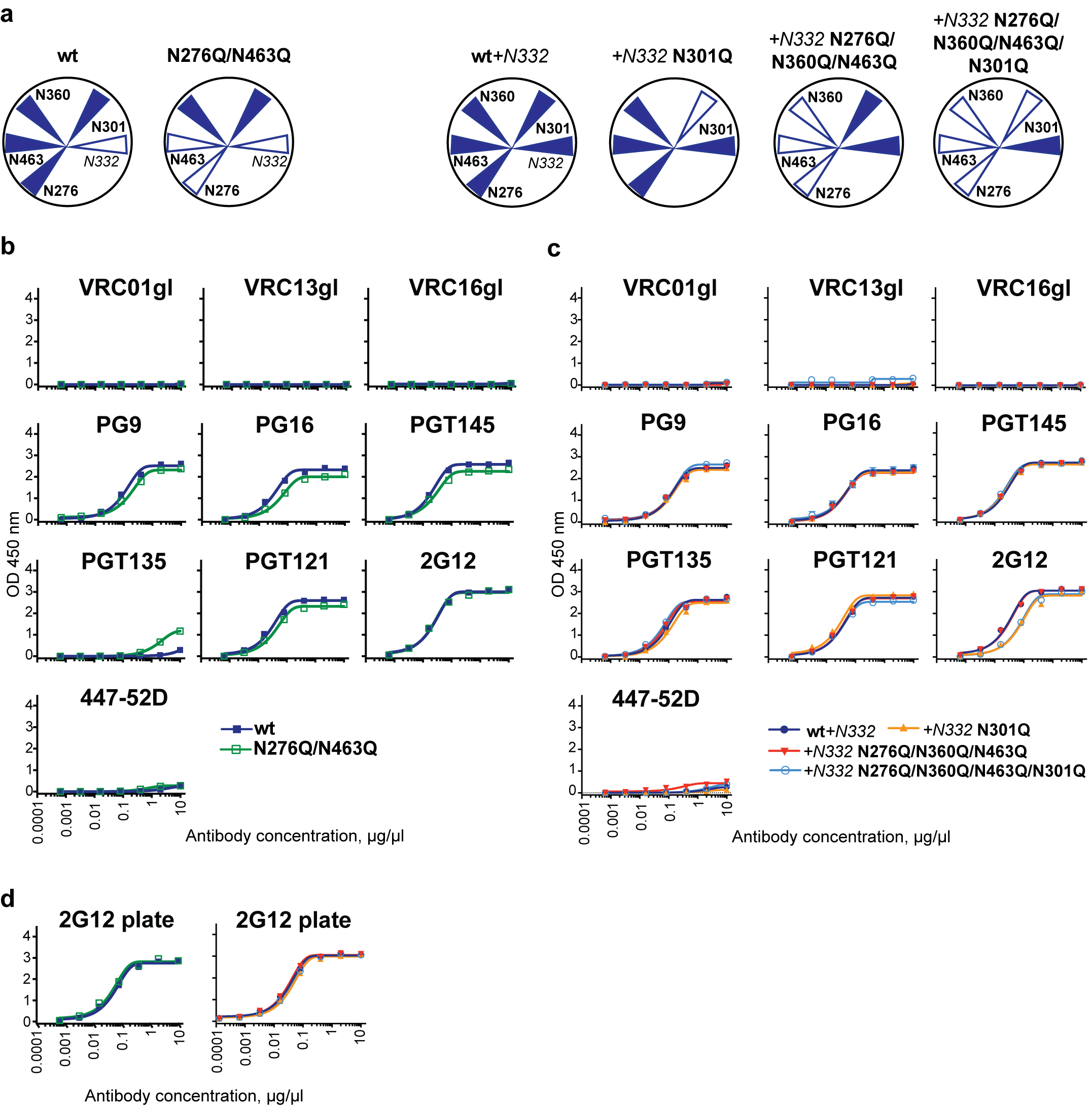

Supplement: S5 Fig — (a) Comparison of the PT (dark blue) and N276Q/N463 (green) trimers. (b) (b) Comparison of the PT (dark blue) and N276Q/N463 (green) trimers. His-captured trimers were analyzed. (c) Comparison of the +N332 PT (dark blue) with +N332 N301Q (yellow), +N332 N276Q/N360Q/N463 (red) and +N332 N276Q/N360Q/N463/N301Q (light blue) trimers. His-captured trimers were analyzed. (d) 2G12 binding of the trimers coated directly on the ELISA plate. Experimental duplicates were analyzed for each antibody dilution, mean values are shown. (TIF) [file ppat.1006614.s005.tif]

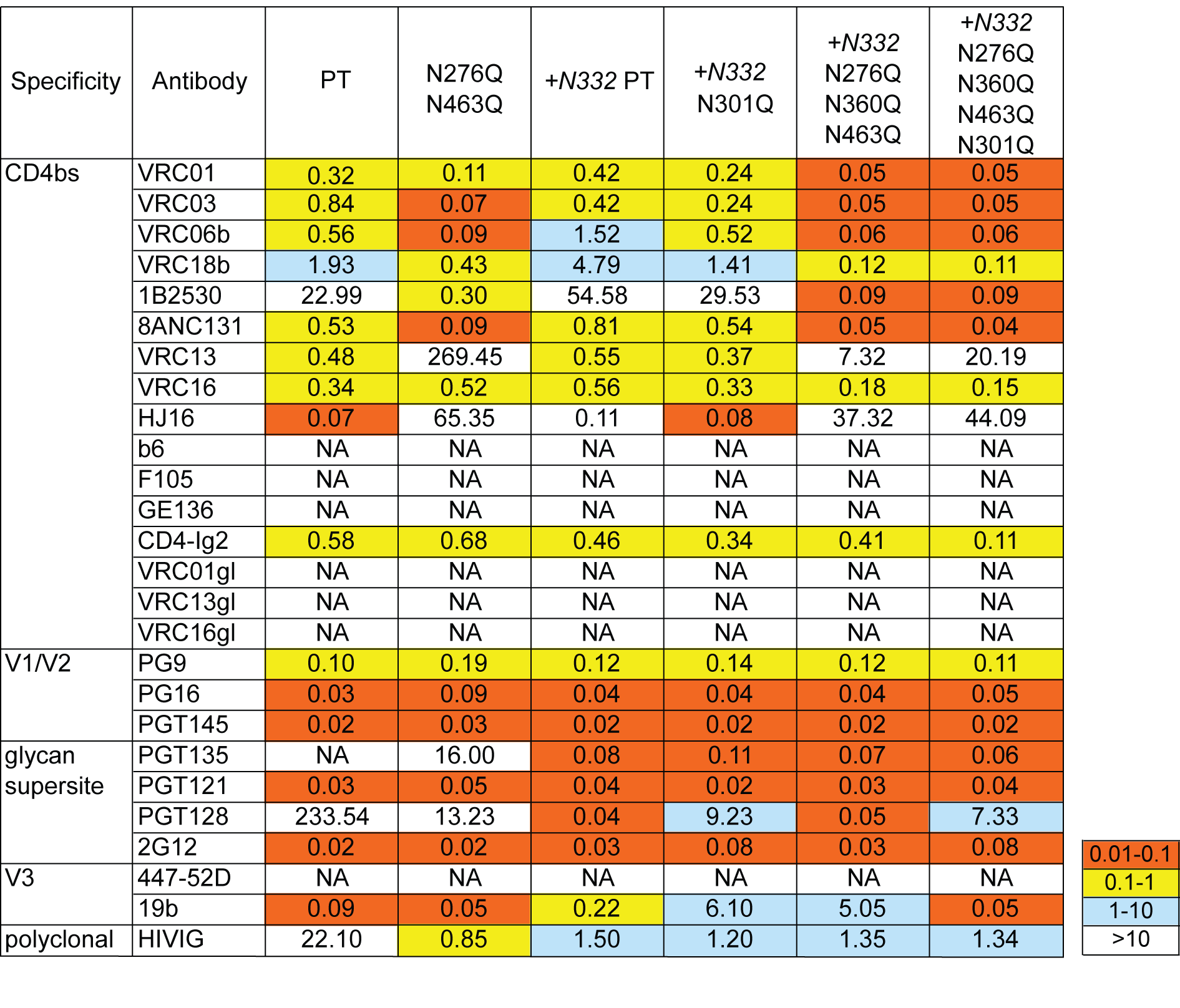

Supplement: S6 Fig — (TIF) [file ppat.1006614.s006.tif]

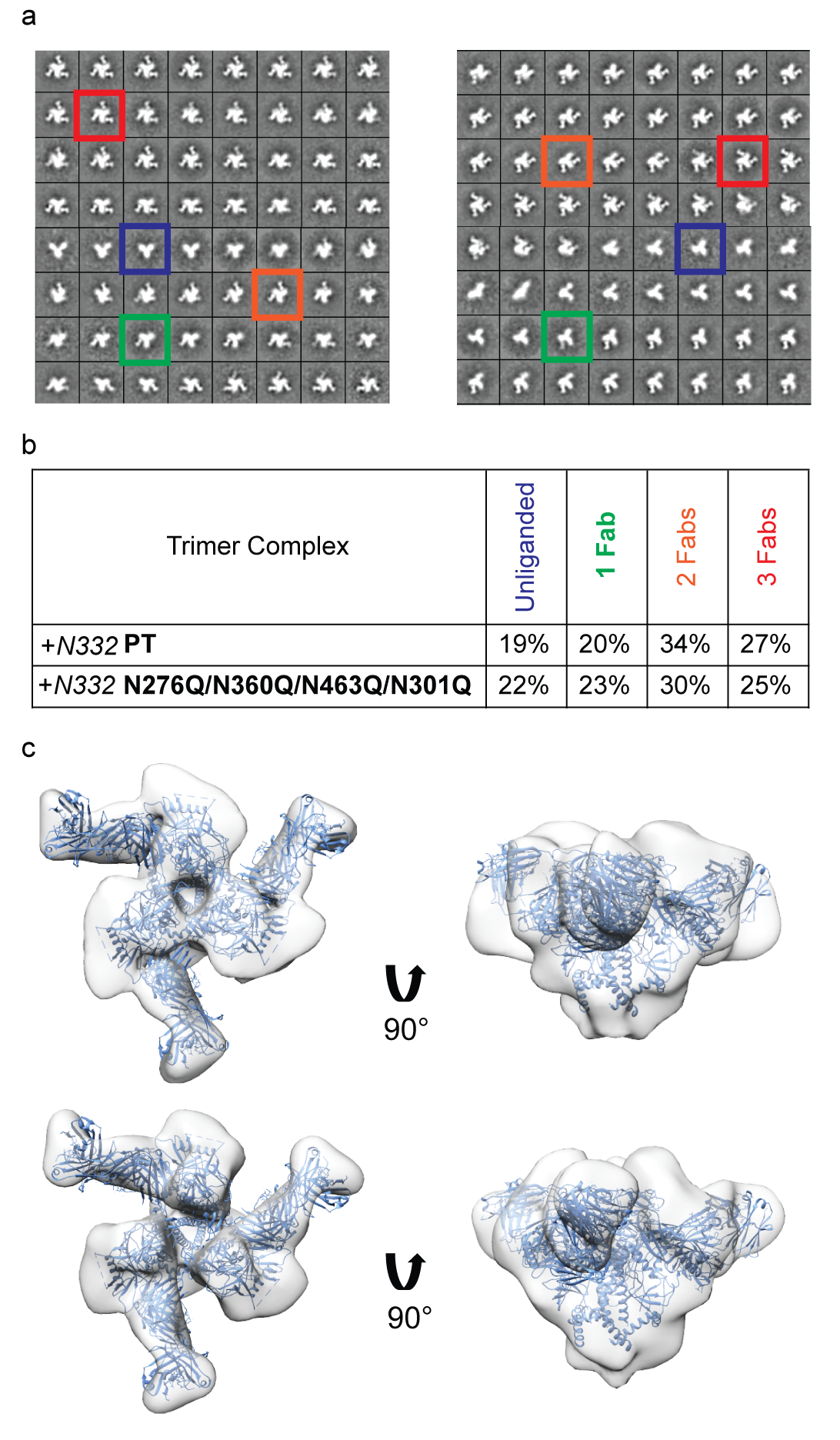

Supplement: S7 Fig — (a) Reference free 2D classes of +N332 PT in complex with VRC03 (left panel) and +N332 N276Q/N360Q/N463Q/N301Q in complex with VRC03 (right panel). Red: 3 Fabs bound, orange: 2 Fabs bound, green: 1 Fab bound, and blue: unbound trimers. (b) Table listing the occupancy of VRC03 Fab relative to the trimers. (c) EM 3D reconstructions of +N332 PT in complex with VRC03 (top panel; symmetry C3 applied) and +N332 N276Q/N360Q/N463Q/N301Q in complex with VRC03 (lower panel; symmetry C3 applied). The crystal structure of the BG505 soluble trimer in complex with PGV04 (PDB:3J5M) was fitted inside the EM volumes. The contour levels used for the symmetric volumes (C3) were ~19. (TIF) [file ppat.1006614.s007.tif]

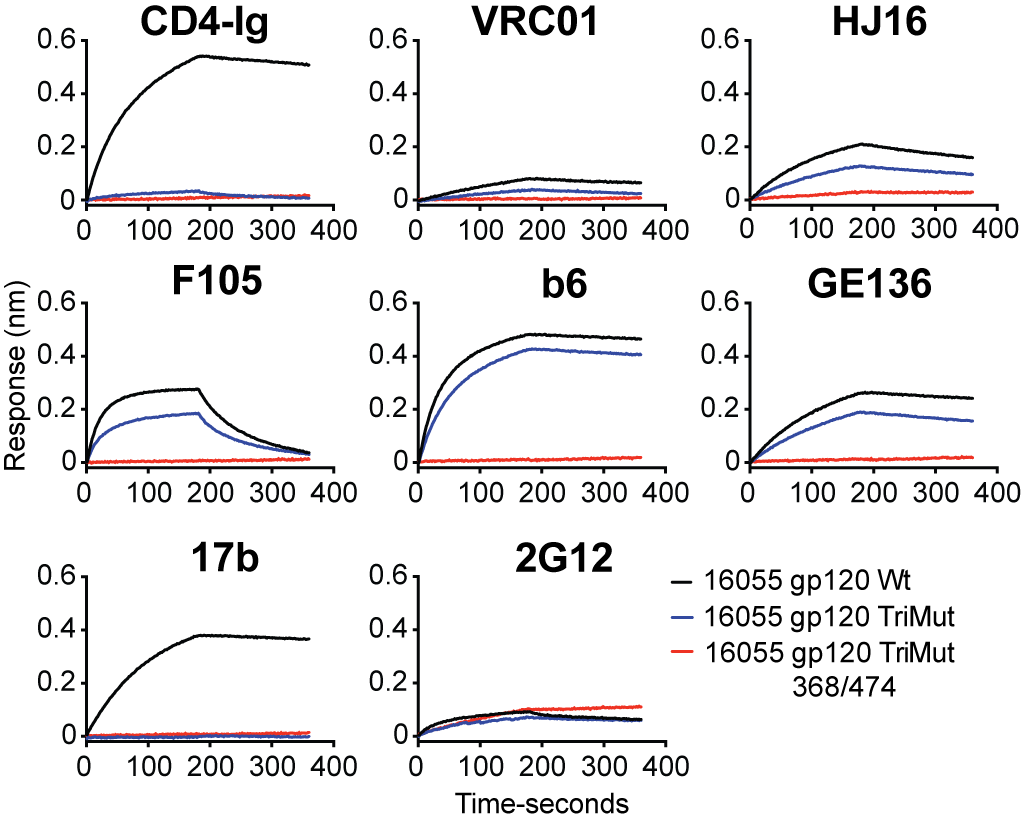

Supplement: S8 Fig — Based on 16055 gp120, two probes, TriMut with triple mutations (I423M, N425K and G431E) and TriMut 368/474 with two additional mutations (D368R and D474A), were designed to map the CD4bs neutralizing antibodies present in sera by neutralization depletion assay. To characterize the binding profile of the probes by Biolayer Interferometry (BLI), a panel of antibodies and CD4-Ig were captured by anti-human IgG Fc sensor and then dipped into 200 nM of probes in the well. The association and dissociation times are 3 min, respectively. (TIF) [file ppat.1006614.s008.tif]

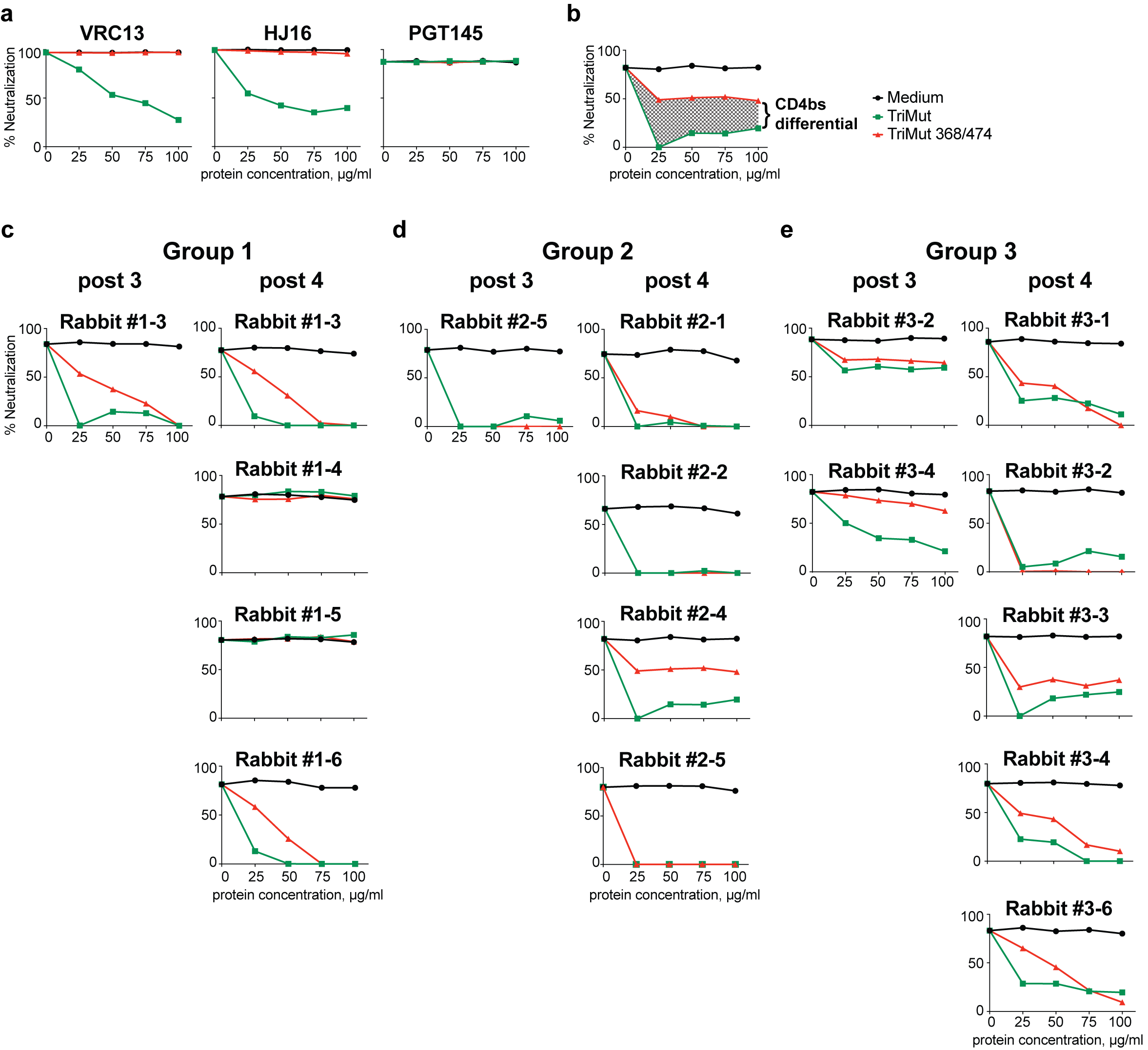

Supplement: S9 Fig — Serum samples with neutralization titers above 100 were used to isolate total IgGs. The purified IgG samples were used in the assay at IC80 concentration. (a) panel confirms the differential depletion capacity of TriMut and TriMut 368/474 probes with CD4bs specific VRC13 and HJ16 bNAbs. PGT145 was used as a negative control. (b) A graphical depiction of the CD4bs differential is shown. Differential assays for Group 1 (c), Group 2 (d) and Group 3 (e) are shown. Two independent adsorption experiments were performed and a representative experiment is shown. (TIF) [file ppat.1006614.s009.tif]
